# Pulsed Electric Field Ablation Reprograms Tumor Immunity and Stimulates Germinal Center Formation in Tertiary Lymphoid Structures in Patients with Non–Small Cell Lung Cancer

**DOI:** 10.1158/1078-0432.CCR-25-3847

**Published:** 2026-06-17

**Authors:** Marcelo Jimenez, Beryl A. Hatton, Alicia Moreno-Gonzalez, Jeffrey S. Iding, Javier Flandes, Erik H.F.M. van der Heijden, Calvin S.H. Ng, Carlos Prieto, Cristina Teodosio, Juan A. Flores-Montero, Marta Rodríguez-González, Oderay Mabel Cedeño Diaz, Shoko Vos, Rainbow W.H. Lau, Medard E. Kaiza, William H. Moore, Alberto Orfao, Tullia C. Bruno

**Affiliations:** 1 Service of Thoracic Surgery, Salamanca University Hospital, Salamanca, Spain.; 2 Salamanca Institute of Biomedical Research (IBSAL), Salamanca, Spain.; 3 University of Salamanca, Salamanca, Spain.; 4 Galvanize Therapeutics, Inc., Redwood City, California.; 5 MedStar Health, Columbia, Maryland.; 6 Hospital Universitario Fundación Jiménez Díaz, Madrid, Spain.; 7 Radboud University Medical Center, Nijmegen, the Netherlands.; 8 Department of Surgery, Prince of Wales Hospital, The Chinese University of Hong Kong, Hong Kong SAR, China.; 9 Bioinformática Service, NUCLEUS, Universitario de Salamanca, Salamanca, Spain.; 10 Translational and Clinical Research Program, Cancer Research Center (IBMCC, CSIC-University of Salamanca), Salamanca, Spain.; 11 Cytometry Service, NUCLEUS, Department of Medicine, University of Salamanca (Universidad de Salamanca), Salamanca, Spain.; 12 CIBERONC Instituto de Salud Carlos III, Madrid, Spain.; 13 Cytopathology Unit, Pathology Department, Salamanca University Hospital, Salamanca, Spain.; 14 Department of Immunology, University of Pittsburgh, Pittsburgh, Pennsylvania.; 15 University of Pittsburgh Medical Center, Pittsburgh, Pennsylvania.; 16 Tumor Microenvironment Center, Hillman Cancer Center, University of Pittsburgh, Pittsburgh, Pennsylvania.; 17 Cancer Immunology and Immunotherapy Program, Hillman Cancer Center, University of Pittsburgh, Pittsburgh, Pennsylvania.; 18 Department of Radiology, New York University Grossman School of Medicine, New York, New York.

## Abstract

**Purpose:**

This treat-and-resect clinical study evaluated a specialized form of pulsed electric field (PEF) ablation’s potential to modulate antitumor immunity in early-stage non–small cell lung cancer (NSCLC). Tertiary lymphoid structures (TLS) are lymphoid aggregates that recruit immune cells into the tumor microenvironment (TME) and serve as immunity-generating neighborhoods. TLS with germinal centers (GC) have strong prognostic value in most cancers and promote tumor control and responsiveness to immune-based therapies, but no commercially available therapeutics induce their formation. The Aliya System is a proprietary electrosurgical technology that delivers microsecond electrical pulses to tissue, achieving tumor clearance while maintaining stromal architecture.

**Patients and Methods:**

Treatment group patients received ablation immediately after diagnostic biopsy versus a biopsy-only control group. Herein, we report on exploratory endpoints examining immune modulation in blood and tumor samples collected from patients in both groups.

**Results:**

Histopathologic assessments revealed the presence of TLS with GC in resected tumors. Tissue cytokine assessments and single-cell RNA sequencing demonstrated upregulation of cytokines capable of recruiting and organizing TLS-associated lymphocytes, including CXCL13, and significant increases in GC-related immune populations, including class-switched memory B and plasma cells in ablated tumors versus preablation biopsies. Ablation group patients had more tumors containing TLS with GC (47%) versus control (24%). Ablation group patients exhibited enhanced systemic immunity, with increased serum high mobility group box 1 and circulating cytotoxic T cells.

**Conclusions:**

This specialized PEF may orchestrate multiple simultaneous changes that promote a more immunologically active TME and represent a new approach for improving immunotherapeutic regimen responses in patients with NSCLC.

**Translational Relevance**

Germinal centers (GC) are paramount for maximal humoral and cellular immunity in secondary lymphoid organs. The presence of GCs within tertiary lymphoid structures (TLS) is associated with improved prognosis and responsiveness to immunotherapy, but no commercially available therapeutics induce their formation. This study demonstrates that a specialized form of pulsed electric field ablation induces tumor cell death and a proinflammatory tumor microenvironment (TME) in the setting of non–small cell lung cancer that favors immune cell infiltration and promotes TLS GC formation and activity. The induced immune composition shift within the TME and systemic circulation includes increased class-switched memory B cells, plasma cells, and activated T cells. Ultimately, these data suggest that this specialized energy may orchestrate multiple simultaneous changes within the TME that result in an immune neighborhood conducive to engaging host immunity against tumors and may therefore represent a new approach for improving responsiveness to or enhancing immunotherapeutic treatment strategies.

## Introduction

Non–small cell lung cancer (NSCLC) is the most common lung cancer, accounting for more than 80% of all diagnoses. Not all patients are eligible for surgical resection, and recurrence and resistance often undermine pharmacologic approaches or treatment with radiation. Currently approved thermal ablation modalities [e.g., radiofrequency ablation (RFA), microwave ablation (MWA), cryoablation] use extreme thermal effects to kill cells, which can result in hemorrhaging, damage to critical structures, loss of tissue/organ function, and efficacy loss in targets near vasculature due to the heat sink effect (1). A significant unmet need, therefore, remains for more effective therapeutic strategies to further improve patient outcomes.

The Aliya System is a proprietary electrosurgical system developed as a minimally invasive technology for ablating tissue via the delivery of specialized pulsed electric field (PEF) energy. The energy is delivered to target tissues via bronchoscopic or percutaneous approaches through a single monopolar needle using conventional navigation techniques. In contrast to thermal ablation, the Aliya System does not rely on thermal gradients to induce cell death, which permits ablation near sensitive structures, including vessels and lymphatics (2, 3).

This specialized PEF energy induces tumor cell death by disrupting transmembrane potentials, resulting in the loss of homeostasis without denaturing cellular and stromal proteins and preserving immunogens and the extracellular matrix (4). Energy delivery subsequently leads to multiple types of cell death, including immunogenic cell death (ICD) mechanisms such as pyroptosis, resulting in the recognition of dying cells by the immune system. Excessive temperatures generated by thermal ablation modalities can result in coagulative necrosis, which can destroy immune cells, denature cytosolic proteins, and preclude immune stimulation and recognition of dying cells (5). In contrast, this form of ablation may allow for a more robust pool of non-denatured tumor antigens. Indeed, ablation with this system has been shown to promote local and systemic antitumor immune activation in preclinical models (4).

Tertiary lymphoid structures (TLS) have strong prognostic value in most solid tumors, including NSCLC. TLS can recruit immune cells into the tumor microenvironment (TME) via cytokine activity (6, 7) and form follicles with germinal centers (GC), which generate memory B cells and long-lived plasma cells that secrete high-affinity antibodies (8), and have been shown to sustain B-cell maturation and antibody production intratumorally (9). Additionally, TLS recruit T cells and bring them into contact with tumor antigens, enhancing antigen presentation and subsequent T-cell activation (10). Consequently, TLS serve as antitumor immunity-generating neighborhoods that promote tumor control and responsiveness to immune-based therapies (10). Recent evidence has shown that the presence of TLS with GC is associated with improved prognosis (9) and therapeutic responses to immune checkpoint blockade (11, 12) in multiple solid tumors. Therefore, strategies aimed at reliably inducing TLS and GC formation with antitumor activity may ultimately be a key therapeutic approach within the setting of cancer.

This study evaluated the capability of this specialized form of ablation to modulate antitumor immunity in patients with NSCLC. Patients with early-stage NSCLC were enrolled in a first-in-human (FIH), two-arm, nonrandomized, multicenter study and received biopsy and ablation or biopsy only prior to surgical tumor resection. The energy was delivered to a solitary, operable lesion during the same anesthetic procedure as the diagnostic biopsy. The intent of this study was not complete ablation coverage, but rather to understand the impact of this specialized energy on the tumor immune parameters. Incomplete ablation was therefore utilized to retain some tumor tissue after ablation.

## Patients and Methods

### Galvanize Aliya System

**Figure 1.**
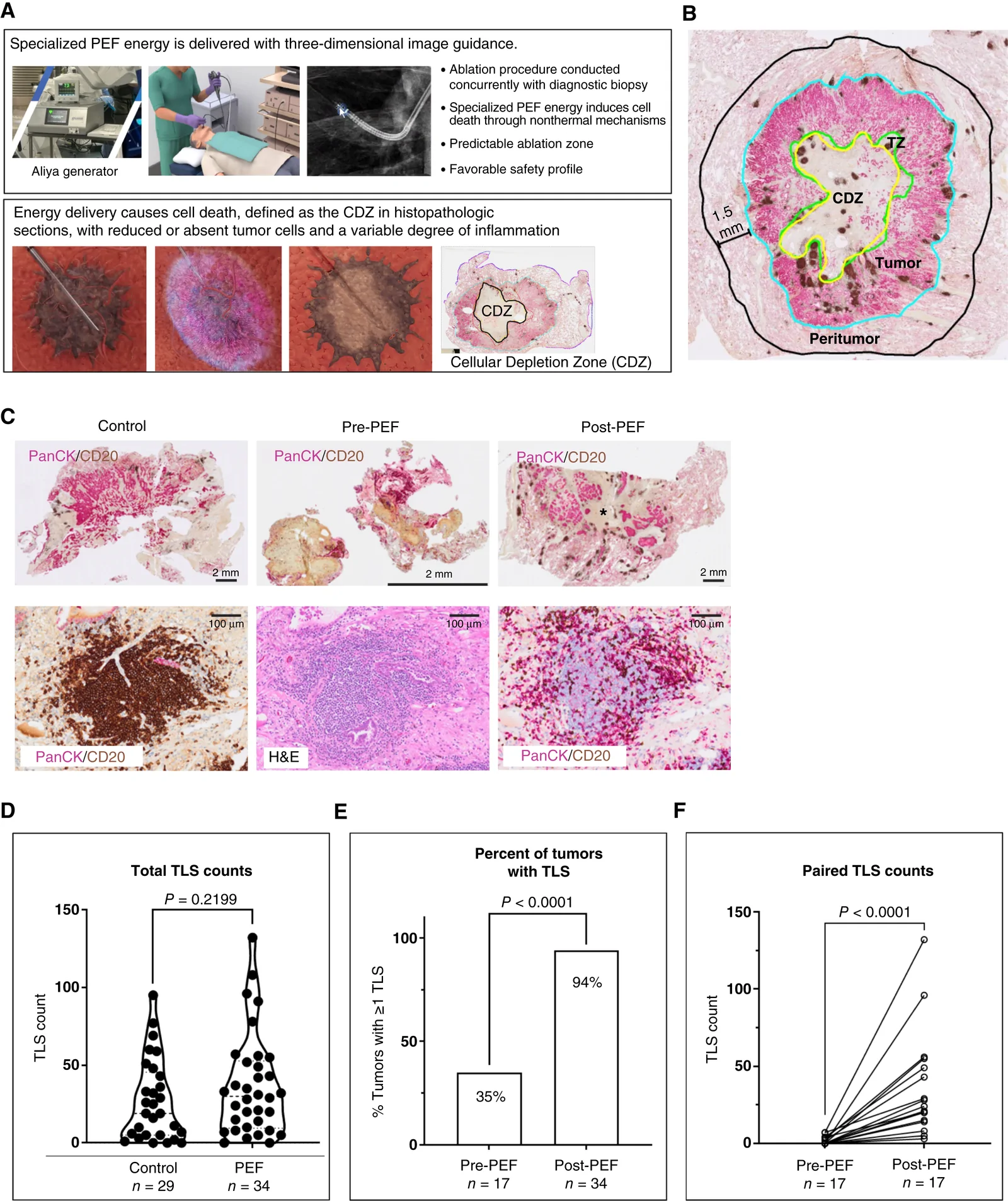
Ablation with a specialized form of PEF energy induces a cellular depletion zone (CDZ) devoid of cancer cells, accompanied by TLS presence. **A,** Ablation needles are placed either via bronchoscopic or percutaneous access with three-dimensional image guidance. Energy delivery results in tumor cell death in the ablation zone. **B,** Multiple ROIs are defined in histopathologic tissue sections collected from resected tumors after ablation, including the CDZ, a transition zone (TZ) extending beyond the CDZ with a greater density of tumor cells but less than those observed in the remaining tumor (RT), and a peritumoral region extending 1.5 mm beyond the invasive margin of the RT. These four regions are collectively referred to as “TLS-relevant zones.” **C,** The intent for this study was incomplete ablation for patients in the ablation group. Patients in the control group received only a biopsy, and tumor resection in both groups was approximately 21 days after the biopsy. Representative images from duplex PanCK/CD20, duplex CD4/CD8, and H&E-stained tissue sections are shown. Top left, A whole-tissue image from the resection sample from a nonablated control tumor; (top middle) an example of a preablation biopsy; (top right) the paired resection sample after ablation. The asterisk (*) denotes the approximate location of the CDZ. Scale bars in the top represent 2 mm. The panels below show a representative example of a TLS evident in a resection sample from an ablated tumor. Scale bars represent 100 μm. **D,** Violin plot showing the total TLS counts by individual tumor for the control (*n* = 29) and ablation groups (*n* = 34). The control group (*n* = 7) was supplemented with additional, nonablated resection samples from patients with characteristics similar to key eligibility criteria who were not enrolled in the study (*n* = 22; see Supplementary Fig. S3). *P* values were calculated using the Mann–Whitney test. **E,** Bar plot demonstrating the percentage of tumors in the ablation group with at least one TLS compared with preablation biopsies. *P* values were calculated using Fisher's exact test. We additionally compared the proportion of TLS-positive tumors before and after ablation in patients with paired pre- and postablation samples (*n* = 17) and found a significant difference between the two time points (*P* < 0.005, calculated using McNemar's χ^2^ test, data not shown). **F,** Plot demonstrating the significant difference in TLS count between pre- and postablation tissue samples from patients with paired samples (*n* = 17). *P* values were calculated using the Wilcoxon signed-rank test. [Created in BioRender. Pastori, C. (2026) https://BioRender.com/4uwlfv7.].

The Aliya® System (Galvanize Therapeutics, Inc.) delivers specialized PEF energy to targeted tissue via either percutaneous or bronchoscopic approaches using three-dimensional imaging [e.g., cone beam computed tomography (CT), augmented fluoroscopy] to confirm needle placement within the target tissue (Fig. 1A). The clinical procedure, system, and energy details were as previously published for the INCITE ES study (2).

### Clinical study design

The INCITE ES study was a prospective, two-arm, nonrandomized, concurrently controlled, multicenter (Spain, the Netherlands, and Hong Kong), open-label, treat-and-resect study (NCT04732520). Results from the primary safety and feasibility endpoints and the full eligibility criteria were previously published (2). This report addresses exploratory endpoints examining immune modulation in blood, serum, and tumor samples collected from patients in both groups. The study was conducted under the authority of the site Ethics Committee, applicable local regulations, International Council for Harmonisation of Technical Requirements of Pharmaceuticals for Human Use (ICH)/FDA Good Clinical Practice (GCP) guidelines, and the Declaration of Helsinki. All patients provided written informed consent prior to their study participation that included consent for their medical data to be used in a study. Between May 2021 and December 2022, the study enrolled 47 patients with suspected or confirmed early-stage NSCLC IA2, IA3, or IB (>1 to ≤4 cm solitary lesion) who were surgical candidates and had no index tumor treatment in the past 2 years. Patient allocation was not blinded, and eligible patients who elected to receive ablation were enrolled in the treatment group. Eligible patients who declined ablation entered the concurrent control group and received standard-of-care (SOC) diagnostic biopsy and surgical resection (Supplementary Fig. S1A). The sample size was based on clinical judgment for a feasibility study and without regard for statistical considerations. Table 1 provides patient demographics, such as age and smoking history, as well as a summary of tumor and procedure characteristics. Supplementary Table S1 describes the representativeness of study participants.

**Table 1.** Patient, tumor, and ablation procedure characteristics (PTE population).

	Ablation (*n* = 34)	Control (*n* = 7)	*P* value
Mean age (range)	67.2 (53–78)	68.6 (55–79)	0.6260
Median age	66.5	68	
Sex (%)	​	​	​
Male	25 (73.5%)	4 (57.1%)	0.3978
Smoking history (%)	​	​	​
Yes	32 (94.1%)	6 (85.7%)	0.2146
NSCLC clinical stage prior to ablation (%)	​	​	0.8745
IA2	16 (47.1%)	4 (57.1%)	​
IA3	9 (26.5%)	2 (28.6%)	​
IB	9 (26.5%)	1 (14.3%)	​
Mean tumor size in cm (range)^a^	2.1 (1–3.7)	1.7 (1–2.3)	0.2249
Median tumor size	1.9	1.7	
Histology (%)^b^	​	​	0.8384
ADC	22 (64.7%)	5 (71.4%)	​
LSCC	9 (26.5%)	1 (14.3%)	​
Other, mADC	3 (8.8%)	1 (14.3%)	​
Energy-delivery/biopsy approach (%)^c^	​	​	>0.9999
Bronchoscopic	24 (70.6%)	5 (71.4%)	​
Percutaneous	10 (29.4%)	2 (28.6%)	​
Mean number of days between biopsy/ablation and surgical resection (range)^d^	21.9 (14–44)	39.7 (19–64)	0.0234
Median number of days	19	35	

a Tumor size is based on the longest diameter prior to energy delivery for the treatment group or prior to biopsy for the control group. There is no statistical significance between groups (*P* = 0.2249).

b Tumors were classified based on the most predominant histologic pattern. For ADCs, specific growth patterns such as solid or acinar were noted, but all were classified under the umbrella of ADC. Mucinous variants were classified separately from primary ADC when a significant mucinous component was present (>60%). This variant was identified by the presence of goblet or columnar cell morphology with abundant intracytoplasmic mucin. LSCCs were grouped together regardless of keratinization subtype.

c Energy delivery for the ablation group, biopsy only for the control group.

d Days between biopsy/ablation and surgical resection differ between study groups (*P* = 0.0234). The control group had a longer interval due to two patients entering the study with prior biopsies (allowed per protocol) and one surgery being postponed due to COVID.

The intent of this study was incomplete tumor ablation to compare the ablated and remaining nonablated tumor tissue following resection. The ablation was adjunctive to the patient’s planned SOC treatment (surgical resection) for their early-stage NSCLC, which occurred within the usual timelines (approximately 21 days from diagnostic procedure to surgical resection). Blood and tumor samples were collected before and after ablation at specific time points (Supplementary Fig. S1B). Sample analyses included histopathologic and single-cell RNA sequencing (scRNA-seq) assessments using tumor tissue, cytokine assessments using serum and tumor tissue, and flow cytometric analysis of peripheral blood (PB), as subsequently described in the specific methods.

The results described herein are from the per-treatment evaluable (PTE) population (Supplementary Fig. S1A; Table 1), which included all enrolled patients who received the ablation or were in the control group, with confirmed NSCLC histopathology after resection and available follow-up data (control group, *n* = 7; treatment group, *n* = 34; Supplementary Fig. S1A). No statistically significant differences were observed in patient age or sex between groups. Tumor size (longest diameter) was not statistically different between the groups prior to biopsy (*P* = 0.2249), with a mean of 2.1 and 1.7 cm in the ablation and control groups, respectively (Table 1). Tumor histologic classification [e.g., adenocarcinoma (ADC), mucinous ADC (mADC), lung squamous cell carcinoma (LSCC)] was determined by study site pathologists.

### Histopathology

Biopsy tissues collected prior to the ablation procedure and surgically resected tumor samples were used for histopathologic analyses. Preablation biopsies with at least 100 tumor cells were included, which represented 17 of 34 patients who received ablation. For postablation resected tumors, CT scans were used as a reference for needle placement within the tumor, and the resected lobe was oriented and cut to locate the tumor. The tumor was then bisected such that gross sections were cut relatively perpendicular to the angle at which the ablation needle was inserted into the tissue for ablation. Gross tissue sections from the resected tumor were formalin-fixed, paraffin-embedded, and processed at a central laboratory (Invicro LLC and Lanterne Dx). The gross tissue section with the most clearly defined ablation zone was selected for IHC assessments. Immunostaining and histopathologic analyses were reviewed and verified by an independent board-certified pathologist. The Visiopharm Integrator System (Research Edition, version 2023.09 or later, Visiopharm Corporation, RRID:SCR_021711) was used to define manual regions of interest (ROI) that distinguished the tumor area from the surrounding nontumor lung parenchyma and annotated regions within the ablation zone as defined in the “Results” section (Fig. 1B; Supplementary Fig. S2). A peritumoral (Peri) region was automatically outlined as a 2 mm margin from the advancing edge of the invasive tumor (i.e., the region extending 1.5 mm beyond the invasive margin; Fig. 1B).

The control group was supplemented with additional resection samples from untreated tumors (*n* = 22) collected from patients who were not enrolled in the study but whose tumors had characteristics similar to key eligibility criteria, including the presence of a solitary NSCLC nodule of stage IA2, IA3, or IB; tumor size; and tumor histologies. These samples were acquired under institutional Ethics Committee approval from Salamanca University Hospital (Salamanca, Spain).

### Brightfield IHC staining and imaging

Gross tissue sections were cut from the block with the most clearly defined ablation zone (ablation group) or tumor bed (control group). From the selected block, serial standard histology sections were stained with hematoxylin and eosin (H&E) and Masson's trichrome or dually stained with antibodies recognizing pan-cytokeratin (PanCK; Abcam, cat. #ab234297, RRID:AB_2895302), CD20 (Abcam, cat. #ab9475, RRID:AB_307267), CD4 (Abcam, cat. #ab213215, RRID:AB_2861280), and CD8 (Abcam, cat. #ab17147, RRID:AB_443686). PanCK stains epithelial-derived cancer cells (carcinomas). Brightfield images from chromogenically stained tissues were captured at 20× magnification using the Olympus automated slide scanner (Evident Scientific, Inc., VS200, RRID:SCR_024783).

### Multispectral IHC staining and imaging

Serial tissue sections from a subset of samples (Supplementary Fig. S3A) were stained with a previously published multispectral immunofluorescence panel (13) using the Opal 6-Plex Detection Kit (Akoya Biosciences, cat. #NEL871001KT, RRID:AB_3674065) as per the manufacturer’s instructions for the sequential staining of each biomarker in the panel. Details for each antibody cycle are provided in Supplementary Table S2. The Opal 6-Plex Detection Kit includes the Opal reagents, secondary horseradish peroxidase (HRP), Akoya Blocking Reagent, and spectral 4′,6-diamidino-2-phenylindole for labeling cell nuclei. Renaissance Background Reducing Diluent (Biocare, cat. #PD905H) was used to reduce nonspecific background staining. Automated multiplex tissue section staining was performed on the Leica BOND Rx autostainer (Leica Biosystems, RRID:SCR_025548). Whole slide scans were captured at 10× magnification using the PhenoImager HT 2.0 platform (Akoya Biosciences, RRID:SCR_023772), followed by spectral unmixing using the built-in algorithm from the PhenoImager HT. Autofluorescence was isolated and removed using an unstained tissue section. This staining was conducted by the Hillman Cancer Center Translational Pathology Imaging Laboratory Core Facility (University of Pittsburgh, RRID:SCR_028073) that is supported in part by award P30CA047904 and the National Surgical Adjuvant Breast and Bowel Project Foundation.

### Brightfield image analysis

The Visiopharm Integrator System (Research Edition, version 2023.09 or later, Visiopharm Corporation, RRID:SCR_021711) was used for digital image analysis. Application-based algorithms were developed to detect structures at the whole slide level and cells within such structures, as defined in Supplementary Fig. S2, using machine learning (deep learning) and thresholding methodology. Images stained for H&E, PanCK/CD20, and CD4/CD8 were loaded and automatically aligned using the tissue alignment module with manual adjustments as necessary (Supplementary Fig. S2). Following tissue annotation in each image using a custom tissue detection deep learning algorithm, manual ROIs were created to distinguish the tumor area from the surrounding nontumor lung parenchyma and to define the ablation zone (Fig. 1B; Supplementary Fig. S2). A trainable deep learning classifier was used to identify immune cell aggregates (ICA) that were >14,000 μm^2^ in size. Criteria for identifying densely contiguous ICA on images from CD20-stained tissue sections were established in collaboration with an independent board-certified pathologist to define a training set. B cell–rich aggregates were initially identified to find potential ICA, which were further reviewed in serial H&E images to confirm follicular characteristics. Two cell phenotyping algorithms were used to count CD20^+^ B cells and CD4^+^ or CD8^+^ T cells, respectively, within the ICA structures. For the detection of individual cells, cells were segmented using a nuclei detection application with custom intensity thresholds to separate the nuclei from the background. The thresholds were selected based on an assessment of the overall staining intensity. CD20, CD4, and CD8 positivity were classified using color deconvolution and phenotyping methodology, with custom intensity thresholds selected for each marker and further optimization using postprocessing approaches. ICAs were considered TLS if they contained ≥50 CD20^+^ B cells and ≥50 T cells (CD4^+^ or CD8^+^) and were >14,000 μm^2^ in size (Supplementary Fig. S3B). We expanded the scoring rubric with an additional deep learning classifier trained to identify GCs in H&E sections within the previously identified TLS (Supplementary Fig. S3B). Training sets for ICA, GC, and nuclei detection, as well as threshold selection and validation output of the trained algorithms, were established and reviewed by an independent board-certified pathologist. The optimized algorithms were then applied to all the images, which were batch processed using the analysis pipeline depicted in Supplementary Fig. S2.

### Multispectral fluorescent image analysis

QuPath (version 0.51 or later, RRID:SCR_018257) image analysis software was used to annotate and quantify the lymphoid structures present in the multispectral images at the whole slide level (14). PhenoImager QPTiff image files were loaded into QuPath and analyzed using a previously published classifier (13). The ROI defining the tumor tissue, peritumoral (Peri) region, cellular depletion zone (CDZ), and transition zone (TZ) was exported from Visiopharm software, converted into GeoJSON files, and overlaid manually onto the multispectral images to align analysis regions. Using QuPath, selected ROIs were randomly sampled from different images and used to train a lymphoid structure pixel classifier. Additionally, cell segmentation using starDist (RRID:SCR_027715; ref. 15) and object classification were trained on the same generated sparse image to maintain generalizability. Following this, a lymphoid aggregate (LA) classifier and object classifier were applied to all images across the multispectral cohort (Supplementary Fig. S3A).

Based on cellular composition, lymphoid structures were categorized into three groups, namely LA, TLS, and TLSs with a GC (TLS_GC). LAs were defined as structures containing at least 15 CD20^+^ B cells, at least 10 CD4^+^ T cells and zero peripheral node addressin–positive (PNAd^+^) cells. TLS were defined as structures containing at least 25 CD20^+^ B cells, at least 10 CD4^+^ T cells, at least one PNAd^+^ cell, and 13 or fewer CD20^+^ activation-induced cytidine deaminase–positive (AID^+^) Ki67^+^ triple-positive cells. TLS_GC met the TLS requirements and included a threshold of more than 13 CD20^+^ AID^+^ Ki67^+^ triple-positive cells within the structure (Supplementary Fig. S3C). Representative examples of the multispectral fluorescent biomarker staining are shown in Supplementary Fig. 4 for TLS (Supplementary Fig. S4A) and TLS_GC (Supplementary Fig. S4B) with the parallel H&E image for reference. For quantification of cell subpopulations, population percentages were generated with the parent population as the denominator (e.g., % CD20:Ki67 cells = total number of CD20:Ki67 dual-positive cells/total CD20-positive cells).

### scRNA-seq

The BD Rhapsody Single-cell Analysis System (BD Biosciences, cat. #633701, RRID:SCR_027096) was used for the simultaneous measurement of cell surface markers (BD AbSeq Immune Discovery Panel, cat. #625970) and mRNA, which enables cell classification based on gene expression and immunophenotype. Tumor tissues obtained from preablation biopsies (*n* = 20) and surgical resection samples (*n* = 25) were used for the analyses. After ablation, core biopsies were collected from regions within the gross ablation zone and the remaining tumor (RT) to ensure that sufficient material was available for sequencing. To obtain cells from the tissue, an enzymatic digestion of the tissue was performed using the specific reagent BD Horizon Dri Tumor & Tissue Dissociation Reagent (cat. #661563) as per the manufacturer’s protocol. Single cells were then labeled with a unique sample barcode conjugated to a human universal antibody (Sample Tag, cat. #633781) to identify the sample, and the pool of cells was labeled with the BD AbSeq Immune Discovery Panel. Cells were then loaded into the BD Rhapsody Cartridge, in which single-cell capture and lysis were performed, obtaining the cDNA from each cell following the manufacturer’s protocol. The cDNA was then used to generate whole transcriptome libraries in combination with the Sample Tag and AbSeq libraries following the Rhapsody WTA AbSeq SMK protocol (BD Biosciences). Sequencing was performed on an Illumina NovaSeq sequencer (RRID:SCR_024568) for a targeted average of 50,000 paired-end reads/cell (150 bp reads).

### scRNA-seq analyses

Raw sequences were demultiplexed with the BD Rhapsody analysis pipeline using the Docker machine version 1.9.1. Resulting read counts were analyzed with R (version 4.3.1 or later, R Project for Statistical Computing, RRID:SCR_001905) and the Seurat package (RRID:SCR_016341; refs. 16, 17) using the human reference version GRCh38. After low-quality cells were removed, data were normalized with the centered log ratio (CLR) method for the AbSeq panel and with the LogNormalize method for the RNA data. Dimensional reduction techniques [principal component analysis (PCA), Uniform Manifold Approximation and Projection for Dimension Reduction, and t-distributed stochastic neighbor embedding] and unsupervised clustering were applied (Leiden clustering). A supervised cell classification and population assignment was performed with Infinicyt Software (BD Biosciences, RRID:SCR_026033) using the AbSeq panel and selected RNA markers (Supplementary Table S3). Differences in cell type proportions were analyzed with the propeller method of the speckle package (18), and *P* values < 0.05 were considered significant. Differentially expressed genes between populations were identified using a Wilcoxon rank-sum test, and *P* values < 0.05 were considered significant.

### Flow cytometry analysis

PB and serum samples were collected at multiple time points, including preablation/biopsy (day 0), days 1 to 5 (early), days 6 to 16 (middle), and days 17+ (late). Data are shown for all available patient samples from the late time point (control group, *n* = 6; ablation group, *n* = 26). Circulating immune cell populations were characterized using a flow cytometry (FCM) panel with 44 cell-surface markers. PB samples were collected in ethylenediaminetetraacetic acid (EDTA) and processed according to the EuroFlow sample preparation and staining standard operating procedures, with some modifications to account for staining of samples with large volumes (www.EuroFlow.org). Briefly, 800 μL of sample were washed 3 times (540 × *g* for 5 minutes) with washing buffer [phosphate-buffered saline (PBS) containing 0.5% bovine serum albumin (BSA), 0.1% sodium azide, and 2 mmol/L EDTA, pH 7.4] prior to staining. Cells were incubated with a viability marker (Live/Dead Fixable Blue, Thermo Fisher Scientific, cat. #L23105, RRID:AB_3717566) in a 1:1,000 dilution for 15 minutes at room temperature, protected from light, and washed with washing buffer (5 minutes at 540 × *g*). Sequential staining with antibodies (Supplementary Table S4) was performed at room temperature and in the dark in the presence of Brilliant Staining Buffer Plus (BD Biosciences, cat. #566385, RRID:AB_2869761). Erythrocyte lysis and cell fixation were performed by incubation with 3 mL of 1x BD FACS Lysing Solution (BD Biosciences, cat. #349202, RRID:AB_2868862) for 20 minutes at room temperature (protected from light), centrifuged for 5 minutes at 540 × *g*, washed with washing buffer, and resuspended in 200 µL of PBS before analysis. Data acquisition was performed on a five-laser (355, 405, 488, 561, and 640 nm) Aurora Cytek flow cytometer (Cytek Biosciences, RRID:SCR_019826) or a CytoFLEX LX (Beckman Coulter Life Sciences, RRID:SCR_027084) instrument. Spectral unmixing was performed by employing single-stained reference controls and each antibody–fluorochrome combination used in the panel, which were processed following the same procedure as the samples. Spectral unmixing was conducted using SpectroFlo software (Cytek Biosciences, version 2.2.0, RRID:SCR_025494), and gating and analysis were conducted using Kaluza analysis software (Beckman Coulter Life Sciences, version 2.2.1, cat. #A84174, RRID:SCR_016182) with previously published gating strategies, as summarized in Supplementary Fig. S5–S7 (19). Assessment of total CD45^+^ cells or total leukocytes showed no significant differences before or after ablation or between the control and ablation groups at each time point collected (Supplementary Fig. S8).

### Cytokine profiling

Cytokine and chemokine concentrations in patient serum and tumor tissues obtained from preablation biopsies and surgical resection samples were analyzed via a 71-analyte Luminex multiplex assay (Human Cytokine Array/Chemokine Array 71-403 Plex Panel, Eve Technologies, HD71). Analytes included CCL1, CCL2, CCL3, CCL4, CCL5, CCL7, CCL8, CCL11, CCL13, CCL15, CCL17, CCL21, CCL22, CCL24, CCL26, CCL27, CD40, CLEC11A, CSF1, CSF2, CSF3, CX3CL1, CXCL3, CXCL5, CXCL9, CXCL10, CXCL12, CXCL13, EGF, FGF2, FLT3LG, IFNA2, IFN gamma (IFNG), IFNL2, IL1a, IL1b, IL1R1, IL2, IL3, IL4, IL5, IL6, IL7, IL8, IL9, IL10, IL12b, IL12p70, IL13, IL15, IL16, IL17A, IL17F, IL18, IL20, IL21, IL22, IL23, IL25, IL27, IL33, LIF, LTA, PDGFA, PDGFB, TGFA, TNF, TNFSF10, TPO, TSLP, and VEGFA. Clinical sites prepared serum samples, and tumor tissue lysates were prepared by Resolian. Azenta Life Sciences shipped sample aliquots to Eve Technologies, where multiplexed reporter antibody cocktails were added to the samples and analyzed on a Luminex instrument to provide a quantitative readout of analyte concentration. Each sample was run in duplicate for each analyte at each time point, and the mean fluorescence intensity (FI) was used for analysis. PCA and ROUT tests were used to identify potential outliers. For the analysis of serum cytokines, no samples were identified as outliers, and samples from two patients were excluded from analysis due to the lack of a baseline (day 0) sample. In total, matched serum samples from 33 patients in the ablation group were analyzed. Serum samples from ablation group patients were normalized to those from the control group to rule out effects from anesthesia or other unrelated effects from the procedure. For the analysis of tissue cytokines, no samples were identified as outliers or excluded, but following the scRNA-seq analysis, tissue only remained from four preablation biopsy samples and 16 postablation resection samples. No tissue remained from the control group. For each patient, the mean log_2_ fold change in FI for each analyte at each time point was calculated relative to day 0, and the mean was then calculated within each group (control, preablation, or postablation) for each time point. *Z*-scores were calculated as follows: (mean within study group and time point − mean across all groups and time points)/(SD across all groups and time points).

### Serum high mobility group box 1

Assessment of serum high mobility group box 1 (HMGB1) was conducted *ad hoc* with remaining samples after planned serum and tissue analyses were conducted. An enzyme-linked immunosorbent assay (ELISA) was used to quantify HMGB1 in serum samples collected at baseline (day 0) and up to day 5 after ablation. Clinical sites prepared and aliquoted serum samples, which were then stored at −20°C prior to shipping. Thawed and diluted serum samples were loaded into a 96-well ELISA microplate coated with an antibody specific for HMGB1 and analyzed according to the manufacturer’s instructions (HMGB1 ELISA Kit, Thermo Fisher Scientific, cat. #EEL047, RRID:AB_3712872). In brief, 100 μL of the standard and each sample were added per well and incubated for 2 hours at 37°C. Each serum sample was run in duplicate. Samples were then removed, and each well was incubated with 100 μL biotinylated anti-HMGB1 antibody for 1 hour at 37°C. One hundred microliters of HRP–avidin conjugate was then added for 1 hour at 37°C. The substrate was then added, and data were analyzed by reading the optical density (OD) at 450 nm using an ELISA reader (Molecular Devices, FilterMax F3) and analysis software (Molecular Devices, SoftMax Pro, RRID:SCR_014789). Duplicate values were averaged, and values were presented as the fold change relative to baseline (day 0).

### Serum immunoglobulin G1

Assessment of serum IgG1 was conducted *ad hoc* with remaining samples after planned serum and tissue analyses were conducted. Human immunoglobulin G1 (IgG1) circulating in patient sera was detected using an ELISA optimized in-house. Clinical sites prepared and aliquoted serum samples, which were then stored at −20°C prior to shipping. Tumor tissue samples were collected during the biopsy procedure performed under image guidance prior to energy delivery. The tissue specimens obtained were immediately snap-frozen and stored at −80°C prior to shipping. Tumor tissue lysates were prepared by Resolian. Fourteen patients in the ablation group had paired serum and biopsy samples remaining, and biopsy-specific IgG1 levels in serum collected prior to the resection procedure were compared with levels in serum collected prior to the ablation procedure (baseline). Briefly, 96‐well microtiter plates were coated overnight at 4°C with 100 μL of proteins extracted from each patient’s biopsy specimen, in triplicates. The following day, the protein lysate was removed from each well, and after three washes with PBS, the wells were subjected to a blocking step with 1% BSA in PBS to prevent nonspecific binding. Paired serum samples were diluted 1:50 in PBS and incubated at room temperature for 2 hours. Next, after removing the sera, the wells were washed extensively with PBS containing 0.05% Tween‐20, and an HRP-conjugated anti-human IgG1 detection antibody (Jackson ImmunoResearch Labs, cat. #109-035-088, RRID:AB_2337584) was applied and incubated for 1 hour. Plates were then washed and incubated with tetramethylbenzidine substrate for 15 minutes, and the reaction was stopped with stop buffer. Absorbance was measured at 450 nm using a microplate reader (Molecular Devices, FilterMax F3) and analysis software (Molecular Devices, SoftMax Pro, RRID:SCR_014789), and the IgG1 levels were quantified by OD. Triplicate values were averaged, and values were presented as the percentage change relative to baseline (day 0).

### Commercial patient selection and data collection

Two patients from a multicenter, Institutional Review Board (IRB)–approved study were included in this article. The study was conducted across five medical centers with data use agreements with each institution and a primary collection site (New York University Grossman School of Medicine, New York). Each institution obtained a waiver of consent from its individual IRB (NYU IRB s23-01036). Lung lesions were ablated with the Aliya System in each patient with the goal of complete coverage. Pertinent clinical data were derived from the electronic medical record of patients referred for ablation who presented with widespread metastatic disease. The ablation procedure was performed via percutaneous access under general anesthesia, with needles placed under three-dimensional CT imaging. Patient follow-up included CT imaging per institutional time intervals.

### Statistical analyses

For all assessments, statistical analyses were performed using GraphPad Software (version 10.2.3 or later, RRID:SCR_002798) or R (version 4.5.2 or later, R Project for Statistical Computing, RRID:SCR_001905), and all tests were two-sided with significance set at *P* < 0.05. For histopathologic analyses, categorical variables are presented as counts and percentages and compared using Fisher's exact test or McNemar's χ^2^ test for paired samples. Continuous variables are presented as medians unless otherwise stated; two independent groups were compared using the Mann–Whitney U test, paired samples using the Wilcoxon signed-rank test, and three or more independent groups using the Kruskal–Wallis test with Dunn’s multiple comparison correction. Area measurements were log-transformed prior to one-way ANOVA with Tukey multiple comparisons test.

For scRNA-seq analyses, differences in cell type proportions were analyzed with the propeller method of the speckle package (18). Differentially expressed genes between populations were identified using a Wilcoxon rank-sum test. *P* values were generated with a Wilcoxon rank-sum test for FCM data and with an unpaired, two-tailed *t* test for cytokine data.

INCITE ES clinical study data were analyzed using SAS software (SAS Institute, Inc., version 9.4 or higher, RRID:SCR_008567). All calculations were based on available data. Per analysis conventions, descriptive statistics and graphical summaries are presented. For categorical variables, counts, percentages, and, where appropriate, *P* values are presented. *P* values were calculated using Fisher's exact test for categorical variables unless otherwise stated. For continuous variables, means, medians, SDs, and 95% confidence intervals (CI) were calculated based on a normal distribution, and *P* values were generated using two-sided, paired *t* tests.

## Results

### A specialized form of ablation induces tumor cell death accompanied by the presence of TLS

The INCITE ES study included two groups: a control group that received biopsy only (*n* = 7) and a treatment group that received biopsy immediately followed by ablation (*n* = 34). Patients in both groups underwent surgical resection approximately 21 days later. Immediately following diagnostic biopsy and intraprocedural malignancy assessment, the specialized energy was delivered intratumorally with three-dimensional image guidance during the same procedural session (Fig. 1A). The demographics, baseline clinical characteristics, and procedure details of the PTE population included in this evaluation are provided in Table 1. The clinical study design is described in the methods, an overview of the study groups is provided in Supplementary Fig. S1A, and a schematic of the patient samples collected is provided in Supplementary Fig. S1B. The safety results for this study have been described previously (2). Briefly, patients tolerated the procedure well; no gross evidence of adverse effects due to the ablation was observed, and the energy delivery had no impact on the planned surgical procedure (2). This, in conjunction with the histopathologic assessment of tissue alterations, indicated that ablation is feasible and safe in NSCLC tumors (2).

Previous histopathologic evaluation of resected samples demonstrated an ablation response with a reduction in malignant tissue proximal to the region where energy was delivered (Fig. 1A and B; ref. 2). This ablation-induced CDZ (Galvanize Therapeutics, Inc.) was characterized by a decrease or absence of tumor cells, accompanied by a variable degree of inflammation, with stromal changes including fibroplasia/fibrosis becoming apparent from tumor cell loss (Fig. 1A and B; Supplementary Fig. S9; ref. 2). A TZ that extended beyond the immediate CDZ demonstrated a greater tumor cell density than in the CDZ but less than that observed in the remaining, nonablated tumor (Fig. 1B; Supplementary Fig. S9).

**Figure 2.**
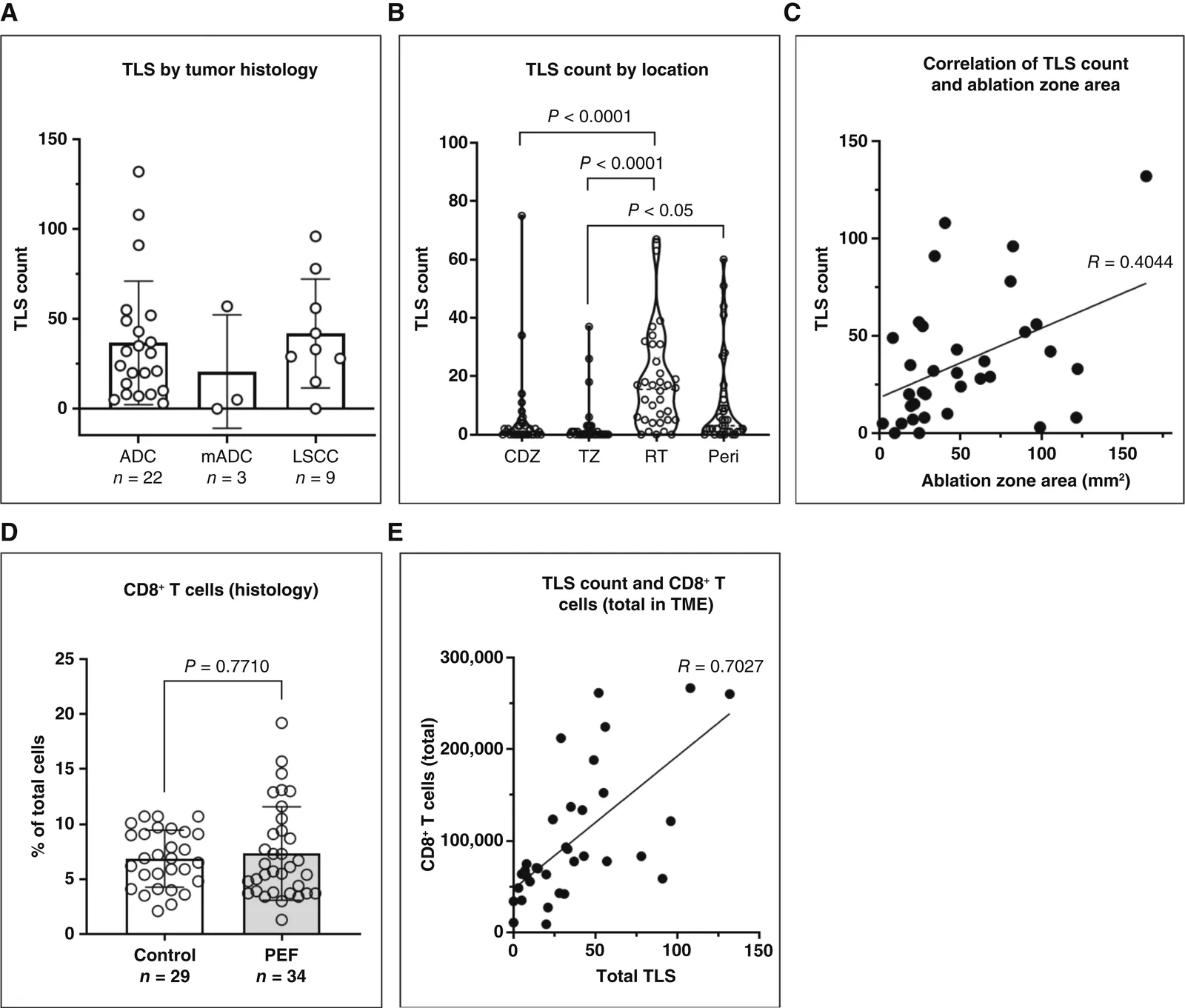
TLS are present regardless of tumor histology and are associated with increased CD8^+^ lymphocytes in the TME. **A,** Quantification of TLS in ablated tumors by histologic subtype [ADC (*n* = 22), mADC (*n* = 3), LSCC (*n* = 9)] shows no difference between histologies (*P* = 0.4653 among the three groups). *P* values were calculated using the Kruskal–Wallis test with Dunn's multiple comparison test. **B,** Violin plots demonstrating the total TLS count per zone in ablated tumors (as depicted in Fig. 1B). TLS were identified within all four TLS-relevant zones (*P* < 0.0001 across the four groups, with significant between-zone differences as specified in the plot), and their accumulation was more robust in the RT and Peri zone. *P* values were calculated using the Kruskal–Wallis test with Dunn's multiple comparison test. **C,** A correlation plot of total TLS with respect to the area in mm^2^ of the ablation zone (CDZ + TZ) indicates a weak positive correlation between total TLS and the ablation zone area (*R* = 0.4044, *P* < 0.05). *P* values were calculated using a nonparametric Spearman correlation. **D,** The percentage of CD8^+^ T cells trended toward being increased in ablated tumors compared with control tumors, as measured in relation to all cells within chromogenically stained tissue sections from each group. *P* values were calculated using the Mann–Whitney test. **E,** A moderate positive correlation was identified between the total TLS count and the total count of CD8^+^ T cells within the TLS-relevant zones in postablation resected tumors (*R* = 0.7027; *P* < 0.0001). *P* values were calculated using a nonparametric Spearman correlation. [Created in BioRender. Pastori, C. (2026) https://BioRender.com/0opxod7.].

Further histopathologic assessments revealed the presence of TLS in ablated tumors (Fig. 1C). Using Visiopharm software, custom image analysis classifiers were trained to identify TLS in brightfield images as defined in Supplementary Figs. S2 and S3. TLS were quantified in a tissue section from each resected tumor, and their location was denoted as being within the CDZ, the TZ, the RT, or within the Peri region. The control group (*n* = 7) was supplemented with additional, nonablated resection samples from patients with characteristics similar to key eligibility criteria who were not enrolled in the study (*n* = 22). Although not statistically significant, an observable trend indicating an increase in the total number of TLS in ablated tumors was noted (Fig. 1D). This trend remained when the analysis was limited to samples from study-enrolled patients (Supplementary Fig. S4C). Quantification from preablation biopsies revealed a significant increase in the percentage of tumors positive for TLS (i.e., containing at least one TLS) following ablation, with 94% of the postablation samples positive for TLS versus 35% of the preablation biopsies (Fig. 1C–E). Analysis of paired biopsy and resection samples (available from 17 of 34 ablation group patients) revealed the same pattern (Fig. 1F). TLS accumulation was observed in ablated tumors regardless of histologic subtype (ADC, mADC, LSCC; Fig. 2A). Intratumoral TLS were observed admixed among tumor cells, along the Peri margin, and within the ablation-induced CDZ and TZ (Fig. 2B). Additionally, a weak positive correlation (*R* = 0.4044) was observed between the total ablation area (CDZ and TZ areas combined) and the total TLS count per tumor (Fig. 2C).

### Energy-induced remodeling within the TME is evident after ablation

**Figure 3.**
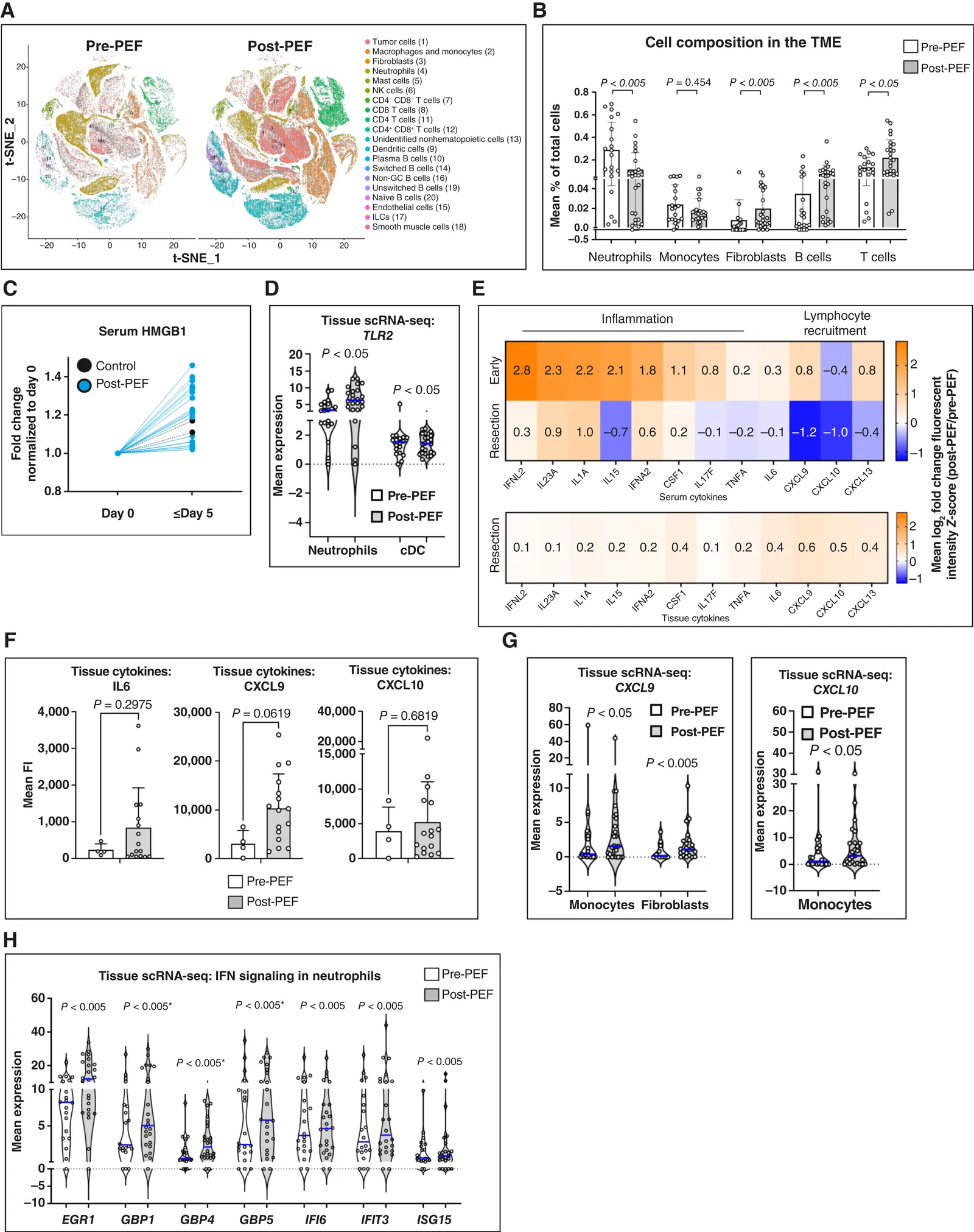
The ablation induces remodeling within the TME that leads to an influx of immune cells. **A,** t-Distributed stochastic neighbor embedding (t-SNE) plots after clustering analysis based on AbSeq and scRNA-seq, showing shifts in gene expression in the various cell populations within the TME (preablation, *n* = 20; postablation, *n* = 25). The annotated subpopulations and classification markers are summarized in Supplementary Table S3. **B,** The mean percentage of total cells is shown (±SD) for each indicated population from patient samples analyzed by scRNA-seq (preablation, *n* = 20; postablation, *n* = 25). *P* values were calculated using the propeller method. **C,** Line graph demonstrating the fold change in HMGB1 levels in serum collected within 5 days after ablation relative to baseline in paired samples from the ablation (*n* = 19) and control groups (*n* = 2). **D,** Violin plots demonstrating the mean expression level of Toll-like receptor 2 (*TLR2*) in neutrophils and cDCs from patient samples analyzed by scRNA-seq (preablation, *n* = 20; postablation, *n* = 25). Blue lines represent the median, and *P* values were calculated using the Wilcoxon rank-sum test. **E,** Heatmap showing the *Z*-score for the mean log_2_ ratio of the fluorescent intensity (FI) cytokine values from postablation samples relative to baseline for serum and tissue cytokines. Cytokines were obtained from serum (*n* = 33), preablation biopsies (*n* = 4), and postablation surgical resection samples (*n* = 16) and analyzed via a multiplexed Luminex assay. Each sample was run in duplicate for each analyte at each time point, and the mean FI was used for analysis relative to baseline levels. Postablation serum samples were collected within 5 days of ablation (early), as well as immediately prior to surgery (resection), and tumor tissue was collected at resection and compared with preablation biopsies. **F,** Bar plots represent the mean FI (±SD) for the indicated cytokines from preablation biopsies (*n* = 4) and postablation samples (*n* = 16). *P* values were generated using a two-tailed, unpaired *t* test. **G,** Violin plots demonstrating the mean expression level of *CXCL9* and *CXCL10* in the indicated cell populations before ablation (*n* = 20) and after ablation (*n* = 25), as analyzed by scRNA-seq. **H,** Violin plots demonstrating gene expression changes in factors involved in interferon (IFN) responses in neutrophils before ablation (*n* = 20) and after ablation (*n* = 25). Asterisks (*) indicate samples for which the adjusted *P* value was also significant (*P* < 0.05). cDC, conventional DC. [Created in BioRender. Pastori, C. (2026) https://BioRender.com/a5lhszw.].

To link immune activity to this specialized energy, we assessed whether the ablation may remodel the TME beyond the localized tumor cell death observed within the CDZ. For this, scRNA-seq was employed to examine changes in the TME from resected ablated tumors compared with preablation biopsies. This analysis revealed cellular composition and gene expression changes in the TME after ablation (Fig. 3A). Notably, significant increases in fibroblasts and B and T lymphocytes were observed in ablated tumors (Fig. 3B). Unlike thermal ablation, in which coagulative necrosis can lead to neutrophil accumulation (20), neutrophil numbers were significantly decreased after ablation. A stress response was evident across many of the cell populations profiled, with increased expression of various heat shock protein (HSP) genes (Supplementary Fig. S10), which help ensure correct protein folding after stress exposure and function in antigen presentation and lymphocyte activation (21). Cell membrane disruption and endoplasmic reticulum stress can lead to damage-associated molecular pattern (DAMP) release (22). In serum samples collected from ablation group patients within 5 days of the ablation, early HMGB1 levels increased relative to preablation and exceeded levels from no-ablation controls in 12 out of 19 (63.2%) patients (Fig. 3C). HMGB1 is a canonical DAMP known to induce ICD when released into the extracellular environment. Toll-like receptor 2 (TLR2) is a pattern recognition receptor that can bind HMGB1, and its expression was significantly increased in neutrophils and dendritic cells (DC) after ablation (Fig. 3D).

Inflammatory serum cytokines were transiently activated within 5 days of ablation and subsequently reduced by tumor resection (Fig. 3E). Only modest increases in these inflammatory cytokines were observed in resected tissue compared with preablation (Fig. 3E). A moderately greater elevation of tissue cytokines linked to lymphocyte recruitment (CXCL9, CXCL10) was observed at resection (23), as was increased IL6 (Fig. 3F). IL6’s functions include promoting T-cell activation, expansion, and survival; promoting B-cell activation and proliferation; and inducing plasma cell survival and antibody secretion (24, 25). *CXCL9* and *CXCL10* gene expression was also significantly increased in monocytes and fibroblasts (Fig. 3G). Notable increases in interferon (IFN) signaling gene expression were also observed, most robustly in neutrophils (Fig. 3H), despite their numbers being decreased after ablation (Fig. 3B). These gene expression changes included enhanced expression of IFN-inducible factors linked with innate immune responses (e.g., *EGR1*, *GBP1*, *GBP4*, *GBP5*, *IFI6*, *IFIT3*, *ISG15*) and increased expression of IFN-inducible genes linked to NLRP3 inflammasome assembly and activation (*GBP1*, *GBP5*; ref. 26).

### Antigen presentation and T-cell activation are accompanied by increased T-cell infiltration

**Figure 4.**
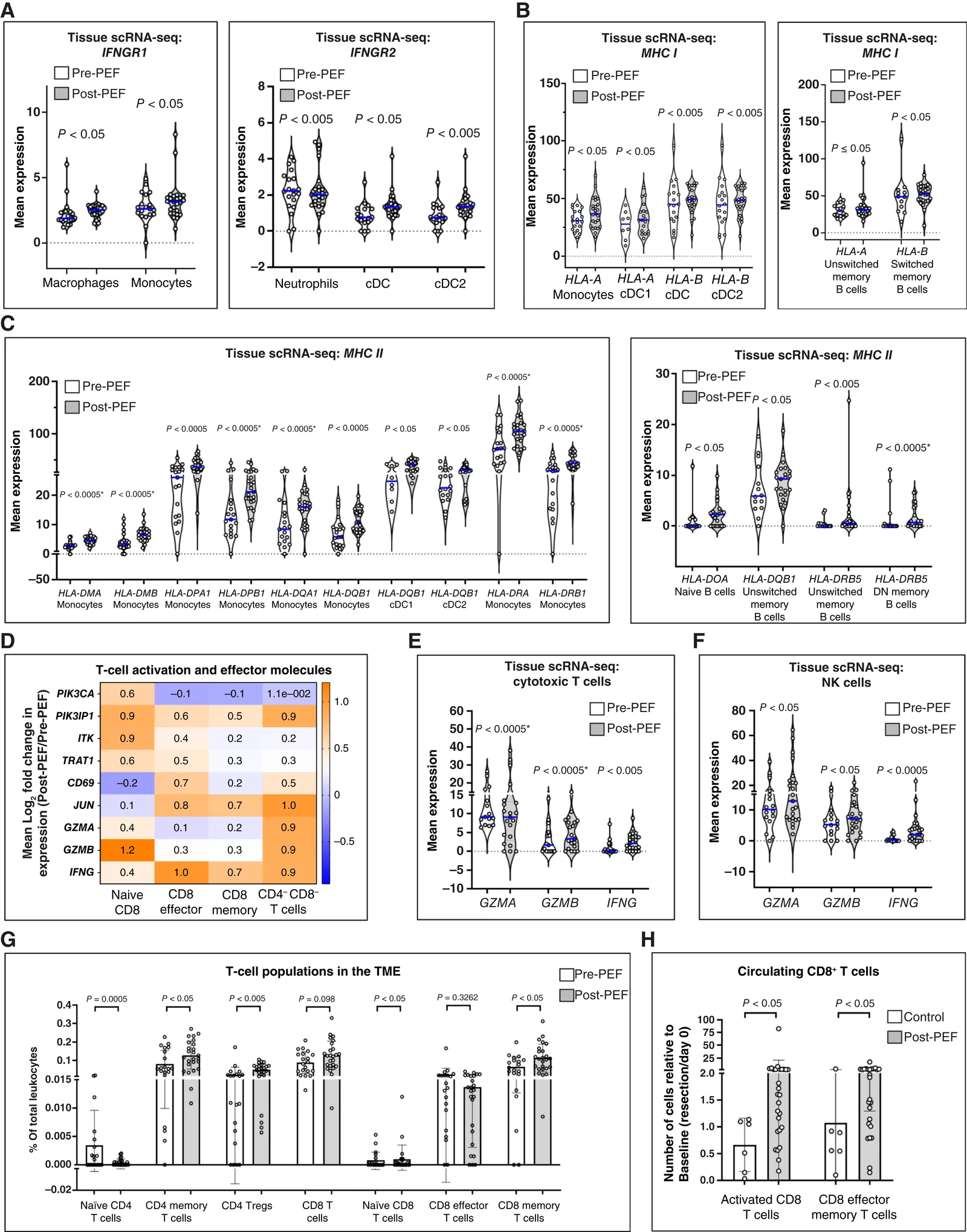
Antigen presentation and T-cell activation are accompanied by increased T-cell infiltration and cytotoxic activity after ablation. **A,** Violin plots demonstrating the mean expression level of *IFNGR1* and *IFNGR2* in the indicated cell populations before ablation (*n* = 20) and after ablation (*n* = 25), as measured by scRNA-seq. Blue lines represent the median, and *P* values were calculated using the Wilcoxon rank-sum test. **B** and **C,** Violin plots demonstrating the mean expression level of various MHC I (**B**) and MHC II (**C**) molecules in the indicated cell populations before and after ablation, as measured by scRNA-seq. Asterisks (*) indicate samples for which the adjusted *P* value was also significant (*P* < 0.05). **D,** Heatmap showing the mean log_2_ fold change in the expression of T-cell activation and effector molecules after ablation (*n* = 25) relative to preablation (*n* = 20) as measured by scRNA-seq. **E** and **F,** Violin plots demonstrating the mean expression level of cytotoxic effector molecules in cytotoxic T cells (**E**) and natural killer (NK) cells (**F**) before ablation (*n* = 20) and after ablation (*n* = 25), as measured by scRNA-seq. **G,** T-cell populations in the TME after ablation, as assessed by scRNA-seq. Bar plots represent the mean percent of each cell population shown relative to the total number of leukocytes in each sample (±SD). *P* values were calculated using the propeller method. **H,** FCM analysis of PB lymphocytes from patients who received ablation and no-ablation control patients. The mean number of cells relative to the day 0 biopsy (preablation in the ablation group) is shown (±SD) from available patient samples (control group, *n* = 6; ablation group, *n* = 26) that were collected 15–30 days after biopsy/ablation. *P* values were calculated using the Wilcoxon rank-sum test. No significant difference in the total number of CD45^+^ cells or the total number of leukocytes was observed before or after ablation or between the control and ablation groups (Supplementary Fig. S8). *GZMA*, granzyme A; *GZMB*, granzyme B; Treg, regulatory T cell. [Created in BioRender. Pastori, C. (2026) https://BioRender.com/napu66a.].

Unlike secondary lymphoid organs such as lymph nodes, TLS are not encompassed by a fibrous envelope, thus enabling contact between immune cells within the TLS and tumor antigens released from dying tumor cells in the TME (27). Although antigen-presenting cells such as macrophages and DCs were not significantly increased after ablation by the time of resection (Supplementary Fig. S11A), they did respond to increased IFN signaling, as evidenced by increased *IFNG* receptor (*IFNGR*) expression (Fig. 4A). IFNGR signaling in DCs can facilitate their maturation, which is the key for priming T cells against tumor antigens. Antigen-presenting genes were also significantly upregulated in monocytes, DCs, and B cells (Fig. 4B and C), as were genes related to antigen experience (*PDIA3*), T-cell activation (*TNFSF9*), and T-cell immune synapse formation (*TUBA1A*; Supplementary Fig. S11B–S11D; refs. 28, 29). T-cell activation and effector molecule expression were elevated in CD8^+^ T cells relative to preablation (Fig. 4D), including granzymes A and B (*GZMA*, *GZMB*) and *IFNG* (Fig. 4E; Supplementary Fig. S11E), which can result from T cells being activated by antigens and can induce the expression of MHC proteins involved in antigen processing and presentation (Fig. 4B and C; ref. 30). Cytotoxic effector activity was also observed in natural killer cells in the TME after ablation (Fig. 4F). CD8^+^ T cells, in general, trended toward increased numbers after ablation, and significant increases were observed in CD4^+^ and CD8^+^ memory T cells (Fig. 4G). Significant increases were observed in circulating activated CD4^+^ T (Supplementary Fig. S11F), activated CD8^+^ T, and CD8^+^ effector memory T cells in ablation group patients compared with control group patients (Fig. 4H), accompanied by a trend toward increased circulating CD4^+^ effector memory T cells (Supplementary Fig. S11F). Notably, circulating CD4^+^ regulatory T cells were significantly decreased (Supplementary Fig. S11F). Additionally, the number of CD8^+^ that infiltrated the TME after ablation trended toward an increase as measured by scRNA-seq (Fig. 4G) or immunostaining (Fig. 2D), with a moderate positive correlation observed between the total TLS numbers and the number of CD8^+^ T cells quantified by IHC within the CDZ, TZ, RT, and Peri regions from ablated tumors (Fig. 2E). Together, these data suggest that TLS presence is associated with increased T lymphocyte infiltration and provide evidence of antigen encounter and local and systemic T-cell activation in response to this form of ablation.

### Ablation-induced remodeling stimulates GC formation and activity

Key signals critical for TLS formation were also detected after ablation, including increased tissue cytokine levels of CXCL13, IL6, and IL7 (Supplementary Fig. S12A). These cytokines are central to B- and T-cell recruitment and organization within lymphoid structures, promoting high endothelial venule (HEV) establishment and GC formation, as well as B-cell and plasma cell development and survival (10). Increased production of CXCL13, together with elevated expression of lymphocyte chemotactic factors, could account for the significant increases in B and T lymphocytes observed within the TME after ablation (Fig. 3B). The histopathologic assessment of resected tumor samples confirmed the absence of encapsulation around the ablated area (Supplementary Fig. S13), as was also observed in preclinical ablation studies, suggesting no barrier was generated in response to this form of ablation that could prevent the observed influx of immune cells (31). Gene expression analysis further revealed significant increases in the expression of genes critical for GC formation and activity (Supplementary Fig. S12B).

**Figure 5.**
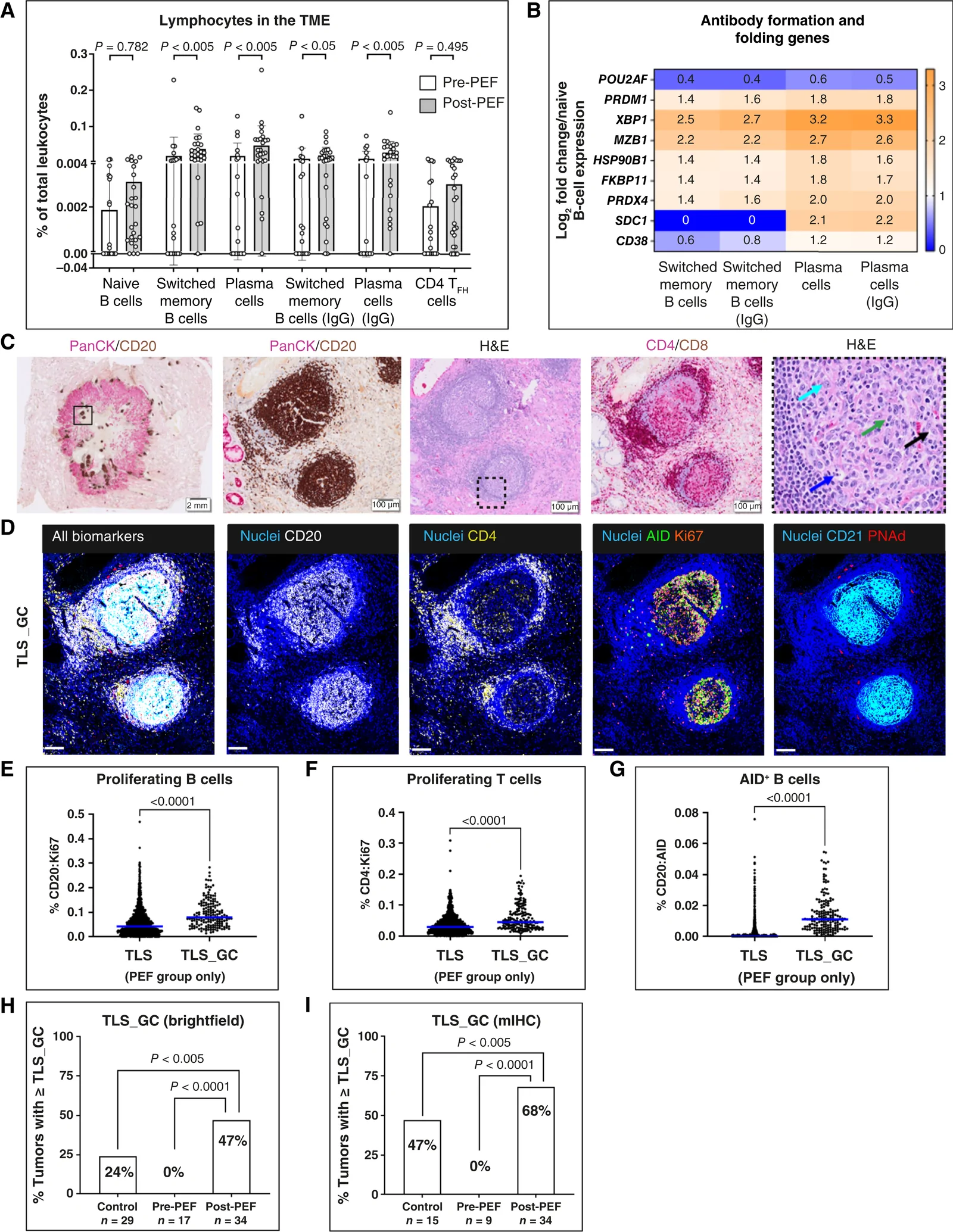
Ablation-induced changes in the TME facilitate TLS and GC formation and functional changes suggestive of GC activity. **A,** B-cell populations in the TME, as assessed by scRNA-seq. Bar plots represent the mean percent of each cell population shown relative to the total number of leukocytes in each sample (±SD). *P* values were calculated using the propeller method. **B,** Heatmap showing the mean log_2_ fold change in the expression of the indicated genes relative to expression levels in naïve B cells as measured by scRNA-seq. **C,** Representative brightfield IHC images of TLS within an ablated tumor after resection. Example images of TLS_GC are shown, with higher magnification images collected from the areas denoted with dashed boxes. The various cell types within a GC are shown in the high magnification panel, including mature B cells within the GC that exhibit the characteristic morphology of proliferating centroblasts (light blue arrow) and centrocytes (dark green arrow). GCs were also found to contain tingible body macrophages (dark blue arrow) and DCs (black arrow). **D,** Multispectral immunofluorescent images from the same TLS_GC in **C**. The first panel shows all biomarkers together [4′,6-diamidino-2-phenylindole (nuclei), CD20, CD4, AID, Ki67, CD21, PNAd], followed by CD20 alone to show B cells, CD4 alone to show T cells, AID and Ki67 together to show proliferating cells and class-switching recombination, and CD21 and PNAd to show FDC networks within the GC and proximal HEV. Scale bars represent 100 μm. **E–G,** Scatter plots of cell frequencies in all TLS_GC compared with TLS in the ablation group (*n* = 34), demonstrating the percent of CD20:Ki67 dual-positive B cells (**E**), CD4:Ki67 dual-positive T cells (**F**), and CD20:AID dual-positive B cells (**G**). Blue lines represent the median, and *P* values were calculated using the Mann–Whitney test. **H,** Bar plot demonstrating the percentage of ablated tumors (*n* = 34) containing at least one TLS_GC from the brightfield image assessment, compared with the preablation biopsies (*n* = 17), as well as to the nonablated tumors from the expanded control group (*n* = 29; *P* < 0.0001 across the three groups, with significant between-group differences as specified in the plot). *P* values were calculated using Fisher's exact test. We additionally compared the proportion of TLS_GC-positive tumors before and after ablation in patients with paired pre- and postablation samples (*n* = 17) and found a significant difference between the two time points (*P* < 0.005, calculated using McNemar's χ^2^ test, data not shown). **I,** Bar plot demonstrating the percentage of ablated tumors containing at least one TLS_GC from the tissue sections stained with the multispectral immunofluorescent biomarker panel (control, *n* = 15; preablation, *n* = 9; postablation, *n* = 34; *P* < 0.0001 across the three groups, with significant between-group differences as specified in the plot). *P* values were calculated using Fisher's exact test. mIHC, multispectral immunohistochemical; T_FH_, T follicular helper. [Created in BioRender. Pastori, C. (2026) https://BioRender.com/79qnysx.].

Further immune cell profiling in the TME via scRNA-seq provided evidence of GC activity, including significant postablation increases in the key cellular outputs of the GC reaction, namely class-switched memory B and plasma cells (Fig. 5A). GC emerge within TLS upon antigen acquisition by resting B cells and their interaction with CD4^+^ T cells, which induces B-cell proliferation, clustering, and differentiation into long-lived memory B or plasma cells that generate tumor-targeting antibodies capable of inducing tumor cell death. This was accompanied by a trend toward increased CD4^+^ T follicular helper cells (Fig. 5A), which are required for GC generation and maintenance (25). Additionally, the expression of antibody production and protein folding genes, as well as genes key to maintaining long-lived plasma cells, was elevated in class-switched memory and plasma cells relative to that in naïve B cells (Fig. 5B), further supporting GC presence and activity.

Ultimately, histologic evaluation of ablated tumors revealed that TLS with GC (TLS_GC) were visible in H&E-stained tissue sections, as evidenced by the presence of organized follicular structures containing mature B cells exhibiting the characteristic morphology of proliferating centroblasts and centrocytes (Fig. 5C). Multispectral IHC staining in a subset of samples (Supplementary Fig. S3) integrated additional biomarkers to confirm the presence of GC within TLS, including AID as a marker of class-switching recombination within B cells, Ki67 (proliferating cells), PNAd to identify HEV, and CD21 as a marker of follicular DCs (FDC; ref. 13). This assessment confirmed that the identified GC contained CD20^+^ B cells dually positive for Ki67 and AID, the presence of HEV (PNAd^+^ cells), and qualitative observation of an FDC network pattern (CD21^+^ cells; Fig. 5D). Additionally, the percentage of CD20^+^ Ki67^+^, CD4^+^ Ki67^+^, and CD20^+^ AID^+^ double-positive cells was significantly different in TLS_GC compared with TLS (Fig. 5E–G), demonstrating functional GC activity in the ablated tumors.

TLS_GC presence was quantified in brightfield images using a Visiopharm classifier validated by a board-certified pathologist (Supplementary Fig. S2). The prevalence of tumors with at least one TLS_GC was significantly increased in ablated tumors (47%) compared with controls (24%; Fig. 5H). These findings were consistent when the analysis was limited to samples from study-enrolled patients (Supplementary Fig. S4D). Similarly, in a subset of samples assessed by multispectral staining, the percentage of TLS_GC-positive tumors remained significantly higher postablation compared with the control group (Fig. 5I). Few TLS were identified in 6 out of 17 preablation biopsies (Fig. 1C–E), and none contained GC (Fig. 5H and I). TLS_GC were found in all ablation zones, and GC size was similar between the CDZ and the RT (Supplementary Fig. S4E). To examine the association of TLS and GC formation with the ablation response, the pathologic response was evaluated within the CDZ. This assessment revealed a major pathologic response (MPR; ≤10% residual viable tumor) in 15 of 22 (68%) samples with an evaluable CDZ. To determine whether TLS and GC formation differed between patients for whom the CDZ reached MPR compared with patients for whom the CDZ did not meet the MPR threshold, the distribution of the total number of lymphoid structures as defined by the multispectral fluorescent IHC (LA, TLS, TLS_GC) was examined in each group (MPR vs. no MPR). The test revealed a statistically significant association between the types of lymphoid structure frequency and patient classification [MPR vs. no MPR; χ^2^ (2) = 50.821, *P* < 0.001], indicating that the distribution of structures was not independent of MPR classification status (Supplementary Fig. S4F). When examining the proportional composition of lymphoid structure types, tumors with a CDZ MPR showed a higher proportion of TLS relative to their total lymphoid structure count, whereas the proportion of TLS_GC structures was more similar between the two groups. These findings suggest that TLS formation might be associated with an improved pathologic response following ablation.

Together, these findings suggest that ablation induces a local proinflammatory microenvironment that favors immune cell infiltration and activation and promotes a humoral immune response via GC formation and maturation within TLS. Serum immunoglobulin (IgG1) levels were elevated in 11 out of 14 (78.6%) ablation group patients with available samples remaining and by more than 25% in three of those 11 patients (Supplementary Fig. S14A). All three patients with greater than 25% elevation in serum IgG1 had TLS with GC present in the resected tumor tissue sample evaluated, as confirmed by the multispectral GC biomarker panel staining. Furthermore, analysis of the percentage of plasma cells (relative to total leukocytes) in the TME, as assessed by scRNA-seq, indicated a positive correlation (*R*^2^ = 0.675, *P* < 0.05) between the percentage of plasma cells present after ablation and the percentage increase in serum IgG1 levels in the patients who had both serum IgG1 and scRNA-seq data available for the analysis (*n* = 7; Supplementary Fig. S14B). These analyses suggest that a systemic response may mirror the immune activation observed in the TME, which was further supported by a trend toward increased switched memory B cells and a significant increase in plasmablasts in PB after ablation (Supplementary Fig. S14C). Together, these findings suggest that the resulting humoral immune response may reach systemic circulation and protect against distant tumor formation.

## Discussion

**Figure 6.**
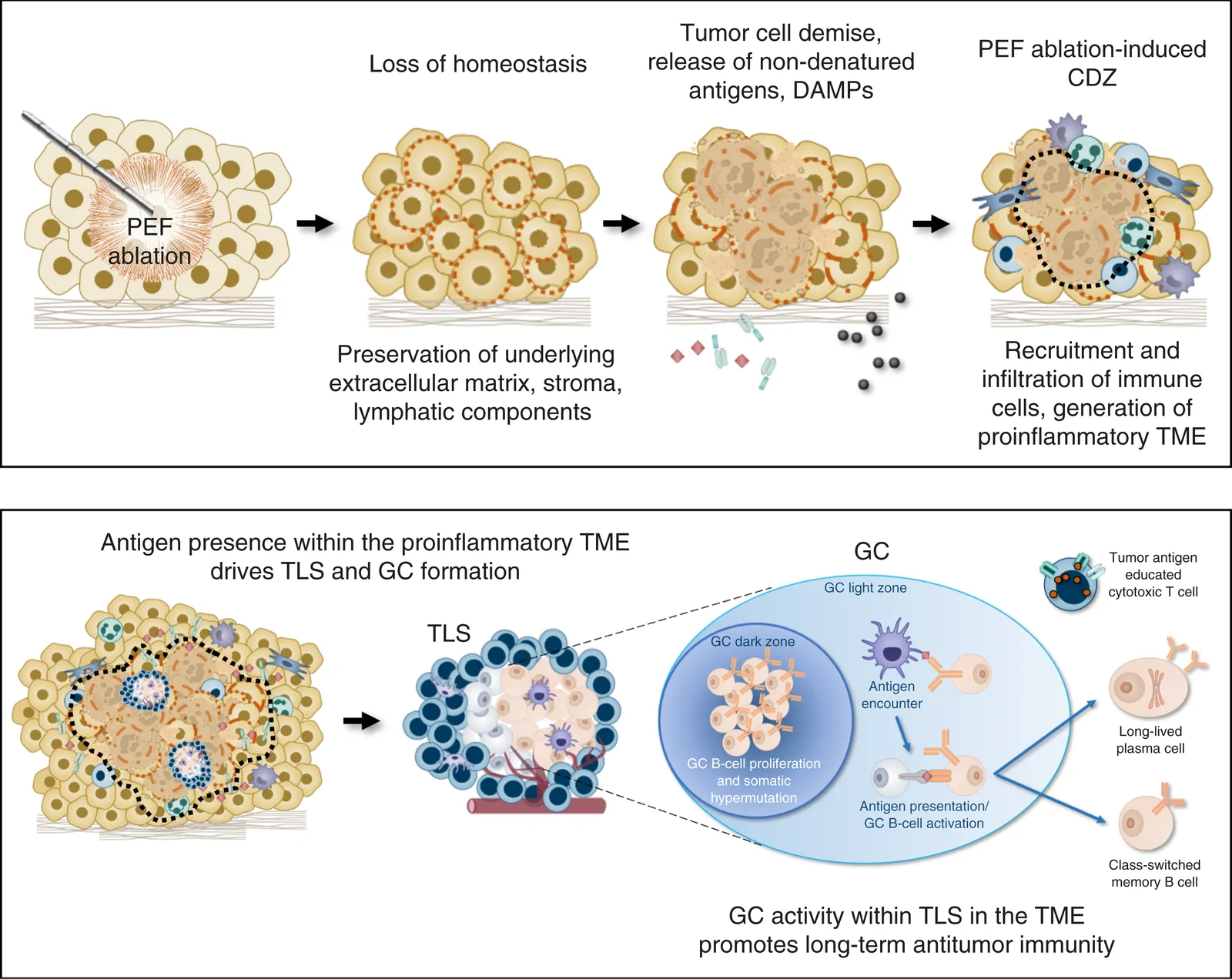
Schematic highlighting the proposed mechanisms of immune modulatory activity induced by the specialized PEF. The Aliya System delivers short-duration, high-voltage electrical pulses that disrupt transmembrane potentials, resulting in a loss of homeostasis, which induces cell death, including ICD, without relying on thermal mechanisms. The resulting injury preserves the underlying extracellular matrix, stromal, and lymphatic components without induction of encapsulation around the ablation area and results in the release of non-denatured tumor-associated antigens and DAMPs as tumor cell death occurs. This loss of tumor cells results in the CDZ, which, together with the release of antigens and DAMPs, induces a proinflammatory state within the TME that subsequently leads to the recruitment and infiltration of immune cells. These ICD processes activate antigen presentation machinery, leading to T-cell priming and activation. Over time, this ICD continues beyond the epicenter of energy delivery, and the presence of antigens in this proinflammatory setting likely leads to the formation and maturation of GC within TLS. The formation of GC within TLS generates class-switched memory B and plasma cells capable of producing antitumor antibodies in the TME or in circulation. Together, these mechanisms may promote a more adaptive, long-term antitumor immunity that helps resolve both local and distant lesions.

This FIH study provides the first clinical evidence that this specialized ablation may have the potential to induce remodeling within the TME and inflammatory changes capable of driving GC formation within TLS and engaging host innate and adaptive immune responses, which may elicit antitumor activity. A schematic highlighting the proposed immune modulatory mechanisms induced by this form of ablation is presented in Fig. 6.

Because the ablation induces cell death without relying on thermal mechanisms, the associated injury results in the release of non-denatured tumor-associated antigens, which may function as potent DAMPs that trigger downstream ICD pathways. Such an ICD response would release proinflammatory cytokines capable of recruiting immune cells into the ablation area, and B and T lymphocytes were significantly increased within the TME following the ablation. Stromal cells have been shown to orchestrate immune infiltration and lymphoid structure formation, and the ablation-induced tumor cell death may have shifted these cells away from a cancer-educated immunosuppressive state (13, 32). Immune cell infiltration into the TME would be facilitated by the preservation of underlying stroma and lymphatic structures in addition to the absence of encapsulation observed around the ablation area. The presence of tumor-associated antigens in this inflamed microenvironment likely further contributed to the observed formation and maturation of GCs within TLS, as well as helper T-cell activation within TLS, together contributing to T-cell education and activation against tumor cells (9, 33). Histologic observations and immune cell profiling of tumor tissue by scRNA-seq demonstrated evidence of GC presence and activity, suggesting these TLS may be educating T cells against tumor antigens and driving B-cell differentiation into memory B and plasma cells capable of generating antitumor antibodies. Increased numbers of activated and memory CD4^+^ and CD8^+^ T cells, switched memory B cells, and plasma cells in the PB of patients, as well as elevated serum IgG1 from the ablation group, further supported the induction of adaptive antitumor immune mechanisms in response to the ablation in the TME and extending systemically. Ultimately, these data suggest that this specialized PEF energy simultaneously orchestrates multiple changes within the TME that result in an immune neighborhood conducive to engaging host immunity against tumor formation.

Although tumor antigen release and immune effects have been observed in response to thermal ablation, reliance upon extreme temperatures results in coagulative cell death, extracellular matrix damage, and immunogenic protein denaturation, which can increase the risk of recurrence and inhibit immunologic tumor responses. Cryoablation maintains cellular structure while permeating cells and leads to the release of DAMPs and inflammatory debris that can have a greater immunostimulatory impact than MWA or RFA (34). However, sublethal temperatures at the cryoablation zone periphery can lead to apoptotic cell death, which can be immunosuppressive and therefore counteractive (35). Furthermore, no evidence of TLS or GC formation has been observed in response to thermal ablation (36).

Immune stimulation via ICD has been observed in response to a subset of cytotoxic agents and radiotherapy (37–40). In addition to DAMP release and immune cell recruitment, radiotherapy can promote the release of tumor-associated antigens that can activate tumor-specific T-cell responses (38). Although abscopal effects have been observed consistently in preclinical studies, clinical observation of nontarget tumor responses in nonirradiated tumors is rare (38). Low-dose radiation alone has been linked to immature TLS formation in mouse models and results in mature TLS when combined with immune checkpoint blockade (39). Only limited observations of TLS induction have been noted clinically in response to other therapies, including cancer vaccines and two small clinical studies showing TLS formation in response to cisplatin or an intratumorally administered CD40 agonist (40, 41). Preclinically, some evidence of TLS formation has been observed in response to immunotherapies such as immune checkpoint inhibitors (ICI), stimulator of IFN genes agonists, a lymphotoxin β receptor agonist, and cytokines, as well as chemotherapies known to induce ICD (40, 42). Although these preclinical findings are promising, the INCITE ES study is the first to demonstrate clear induction of GC formation and maturation within TLS in humans in the setting of NSCLC and highlights how immune modulation induced by this specialized PEF energy may lead to improved responses in patients receiving immunotherapy.

These immune modulatory benefits could potentially extend beyond the ablated tumor and into systemic circulation to protect against distant tumor formation, a hypothesis supported by the abscopal responses noted in preclinical studies and case studies from the commercial use of the Aliya System (33, 43–45). Preclinical studies have demonstrated that this specialized PEF, in combination with immune checkpoint blockade, eliminated unablated lung metastases (4). The combination of this ablation with chemoimmunotherapy enhanced local tumor control, resolved preexisting metastases, and extended survival in a murine model known to be poorly infiltrated by immune cells (46). Abscopal responses have also been noted in clinical cases from early adopters using the commercially available Aliya System in patients with metastatic disease (43–45), and two examples from patients with stage IV disease that had progressed through prior therapy are shown in Supplementary Fig. S15. Additionally, significant improvements in progression-free survival and overall survival associated with this specialized PEF when added to SOC therapy in the setting of stage IV NSCLC after progression on one or more lines of systemic therapy compared with a matched cohort (47) further support that the ablation may represent a locally delivered therapeutic option that could adjunctively sensitize patients to immune checkpoint blockade or enhance control of metastatic disease.

TLS presence confers strong prognostic value and serves as a biomarker for longer-term outcomes and response to checkpoint inhibitor blockade in many solid tumor types (40). Some contradictory findings have been reported, but recent studies taking into account the presence of GC within TLS have shown a consistent association with improved prognosis (40). Our study provides evidence suggesting that GC formation within TLS occurs in response to this form of ablation in the setting of NSCLC and is associated with an increased prevalence of class-switched memory B cells, plasma cells, and activated T cells in the TME and circulating systemically. This shift in TME immune composition has important implications for improving responsiveness to immunotherapies. T-cell infiltration within the TME has been shown to be critical for ICI efficacy (48), and intratumoral TLS have been shown to correlate with T-cell infiltration into tumors (49, 50). The increased presence of TLS_GC and infiltration of T cells after ablation, therefore, suggests that this approach may improve responsiveness to or enhance immunotherapeutic treatment strategies. Further studies are warranted to examine this potential.

Although TLS can form in response to chronic inflammation caused by pathologic conditions such as infections or autoimmune disease, their presence in and around the ablation zone suggests they may have formed in response to the tumor antigens released and the local inflammation induced by the energy. The significant increase in tumors positive for TLS_GC provides further evidence that the immune modulatory activity induced by the energy contributes to adaptive immune responses in the TME. Although the reported incidence of TLS and TLS_GC varies considerably in the published literature, the observed TLS_GC frequency in 47% of tumors following this form of ablation was also greater than a previously reported prevalence of 7% in early-stage, treatment-naïve NSCLC (51, 52). Furthermore, this 23% increase in ablated versus the untreated control tumors was greater than the observed 12.5% increase in TLS_GC noted in a stage II-IIIA cohort of resectable patients with NSCLC treated with chemoimmunotherapy versus a treatment-naïve group (53). Our findings additionally suggested that TLS and GC formation may be associated with an improved pathologic response following ablation. The correlation between pathologic response and overall survival has been demonstrated in multiple solid tumors, including resectable NSCLC following neoadjuvant chemoimmunotherapy, which would suggest that the increased prevalence of TLS in this study may be a positive indicator of treatment response to the ablation and improved longer-term outcomes (54, 55).

Study limitations include the intentionally incomplete ablation necessary for comparing ablated and unablated tissue from the same specimen. Although incomplete ablation enabled the assessments described herein, it is critical to note that the current clinical use of the commercial Aliya System aims to achieve full tumor coverage whenever clinically feasible. Additional study limitations include the smaller size of the control group, the smaller physical size of the preablation biopsy tissue compared with the resection sample (which may not be fully representative of TLS presence at baseline), the single postablation time point at which immunologic changes were assessed via histology, and the limited follow-up time frame that precludes comparing TLS and immune cell characteristics with longer-term patient outcomes. Additional bias could have been introduced by the limited tissue sampling necessary to facilitate multiple assessments from each resected sample (tissue cytokines, scRNA-seq, and histopathologic evaluation); samples collected may not have fully reflected the immune and genetic heterogeneity present within individual tumors. Ongoing clinical investigations are examining the energy’s safety and efficacy for use as neoadjuvant therapy for soft tissue ablation in resectable NSCLC (NCT05583188) as well as in patients with metastatic lung cancer prior to SOC treatment (NCT05890872). The latter includes a secondary endpoint to profile circulating immune cells (56). Future studies will continue to evaluate efficacy in a broader range of solid tumors, including potential systemic antitumor activity and evaluating larger ablation zones.

## Supplementary Material

Supplementary Figures S1-S15 Supplementary Figure 1. CONSORT diagram of patients in the INCITE ES study (NCT04732520) and study schematic. Supplementary Figure 2. Brightfield image analysis pipeline. Supplementary Figure 3. Samples included in the brightfield IHC and multispectral fluorescent IHC analyses Supplementary Figure 4. Comparison of TLS and TLS_GC multispectral immunohistochemistry biomarkers, TLS quantification results from INCITE ES sample groups, and the association of TLS and GC formation with the pathological response to ablation. Supplementary Figure 5. Gating strategy for identification of CD4+ T cell populations by flow cytometry Supplementary Figure 6. Gating strategy for identification of CD8+ T cell populations by flow cytometry Supplementary Figure 7. Gating strategy for identification of B cell populations by flow cytometry. Supplementary Figure 8. Analysis of peripheral blood samples by flow cytometry. Supplementary Figure 9. Histopathological example of the ablation-induced cellular depletion zone (CDZ). Supplementary Figure 10. Ablation induces increased expression of stress response genes. Supplementary Figure 11. Ablation induces increased expression of genes involved in antigen recognition and T lymphocyte activation as well as increased activated and memory T lymphocytes in circulation. Supplementary Figure 12. Genes critical for GC formation and activity are significantly elevated post-ablation Supplementary Figure 13. Ablation does not result in encapsulation around the CDZ. Supplementary Figure 14. Ablation induces a humoral immune response that results in increased serum IgG1, switched memory B cells, and plasmablasts in circulation. Supplementary Figure 15. Off-target responses observed in non-ablated lesions distant from those ablated with the Aliya System in patients not part of the INCITE ES study.

Supplementary Table S1 Representativeness of Study Participants

Supplementary Table S2 Multispectral Immunofluorescent Antibodies

Supplementary Table S3 Single-Cell RNA Sequencing Cell Classification Scheme

Supplementary Table S4 Flow Cytometry Panel Antibodies

## Data Availability

Written requests with methodologically reasonable proposals for access to study data will be reviewed and may require data use agreements. Any data provided will be deidentified and in compliance with applicable privacy laws as well as data protection, consent, and anonymization requirements. Requests should be directed to Galvanize Therapeutics at info@galvanizetx.com. Analyzed scRNA-seq data have been deposited in the NCBI Gene Expression Omnibus under accession GSE325414. Raw data have been deposited in the Sequence Read Archive under accession PRJNA1435204. The code for the scRNA-seq data processing and analysis is available via GitHub: https://github.com/BioinfoUSAL/Hatton-et-al.
