# Supplementary material for: Pulsed Electric Field Ablation Reprograms Tumor Immunity and Stimulates Germinal Center Formation in Tertiary Lymphoid Structures in Patients with Non–Small Cell Lung Cancer
Source: Clin Cancer Res. 2026 Jun 17;32(17):3830–48. doi: 10.1158/1078-0432.CCR-25-3847 (PMC13530995; doi:10.1158/1078-0432.CCR-25-3847)
Supplement: Supplementary file 1 [file ccr-25-3847_supplementary_figures_s1-s15_suppfs1-fs15.pdf]

Pulsed Electric Field Ablation Reprograms Tumor Immunity and Stimulates Germinal Center Formation in Tertiary Lymphoid Structures in Patients with Non-Small Cell Lung Cancer

Supplementary Materials

Fig. S1 to S15

References

Figures

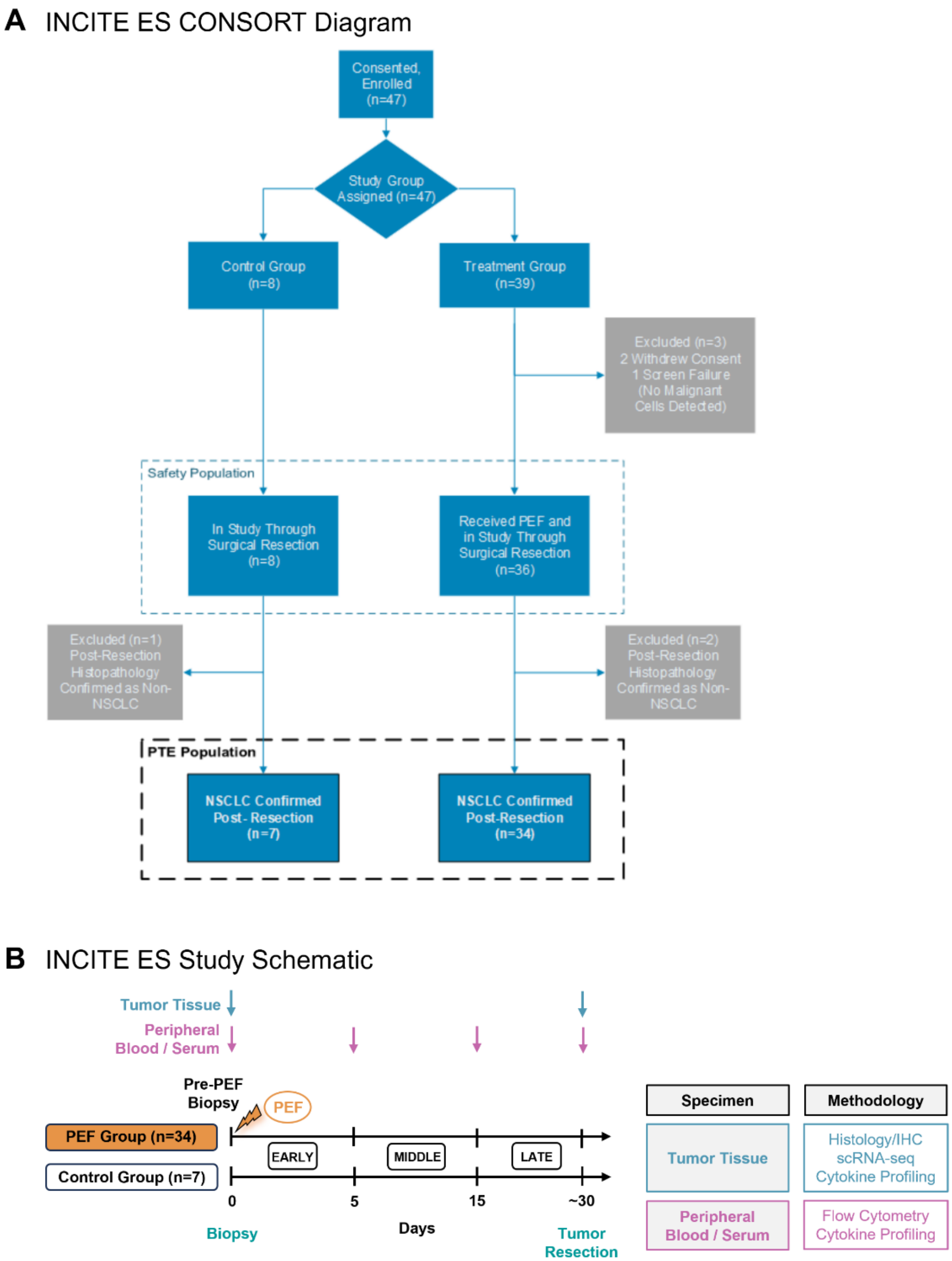

Supplementary Figure 1. CONSORT diagram of patients in the INCITE ES study (NCT04732520) and study schematic. (**A**) This evaluation included patients from the Per-Treatment Evaluable (PTE) population. The PTE population included two groups: a control group that received

biopsy and surgical resection (n=7), and an ablation group that received biopsy immediately followed by ablation and surgical resection (n=34). Enrolled patients underwent a standard of care diagnostic biopsy and intraprocedural malignancy assessment. Patients in the ablation group then received ablation. Seventeen of the 34 patients in the ablation group had paired pre-ablation and post-ablation tumor samples. (**B**) For patients enrolled in the INCITE ES study, tumor tissue was collected on the day of diagnostic biopsy (pre-ablation) and on the day of resection (a mean of 21.9 days post-ablation). Peripheral blood and serum were collected as indicated by the arrows in the schematic. Patients in the control group received a biopsy on day 0 but no ablation, and all other samples were collected as in the ablation group. (Created in BioRender. Pastori, C. (2026) https://BioRender.com/tb6plsi).

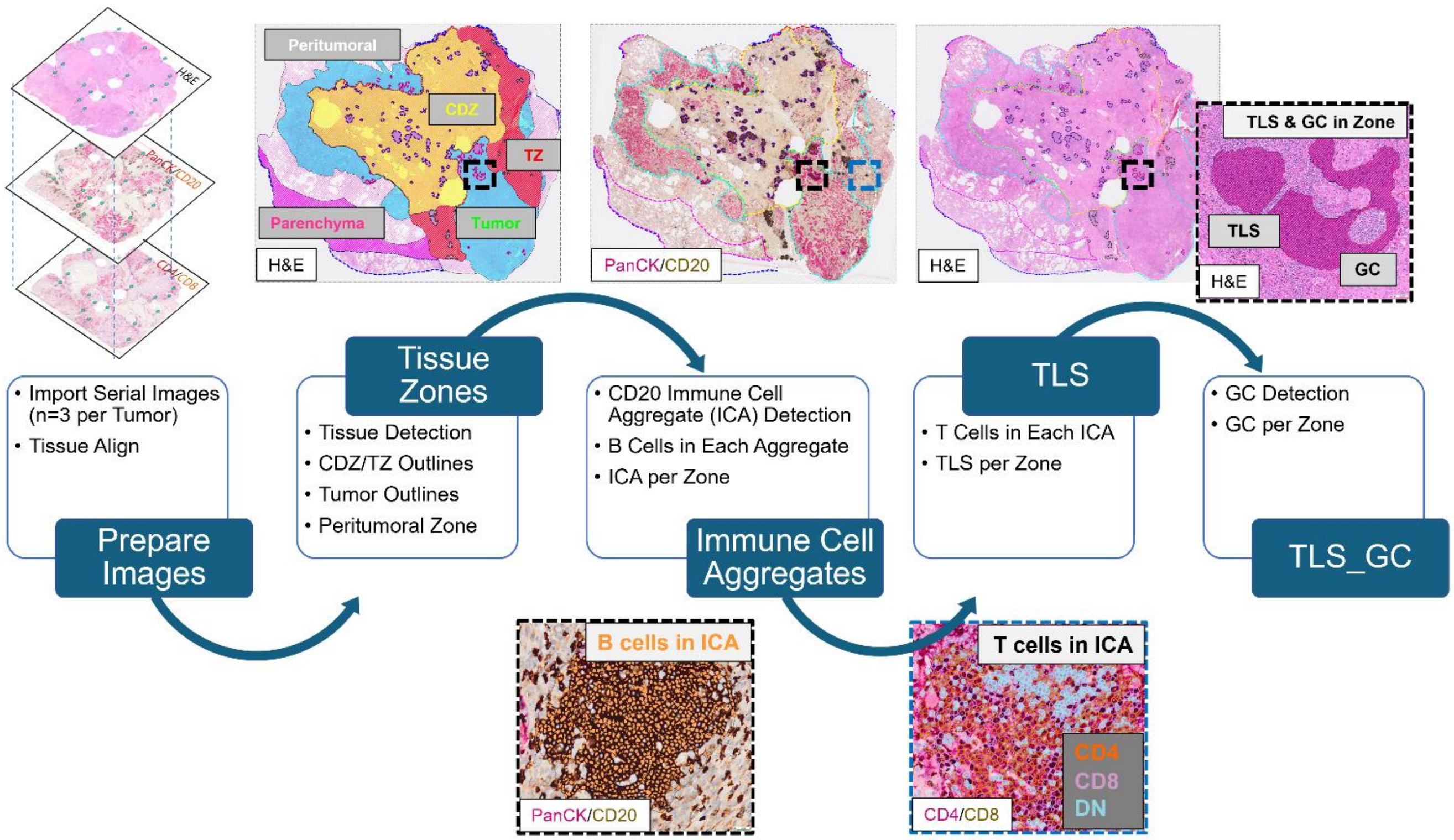

Supplementary Figure 2. Brightfield image analysis pipeline. (**A**) Overview of the Visiopharm digital image analysis process using machine learning algorithms trained and validated with board-certified pathologist input. Using Visiopharm image analysis software, brightfield images of serial tissue sections stained with duplex PanCK/CD20, duplex CD4/CD8, and H&E were aligned, and tissue zones (defined in **Figure 1B**) were outlined in a single slide per patient. Classifiers were trained to identify B cells ($CD20^+$), T cells ($CD4^+$ or $CD8^+$), $CD20^+$-rich lymphoid structures, and GC (H&E).

A

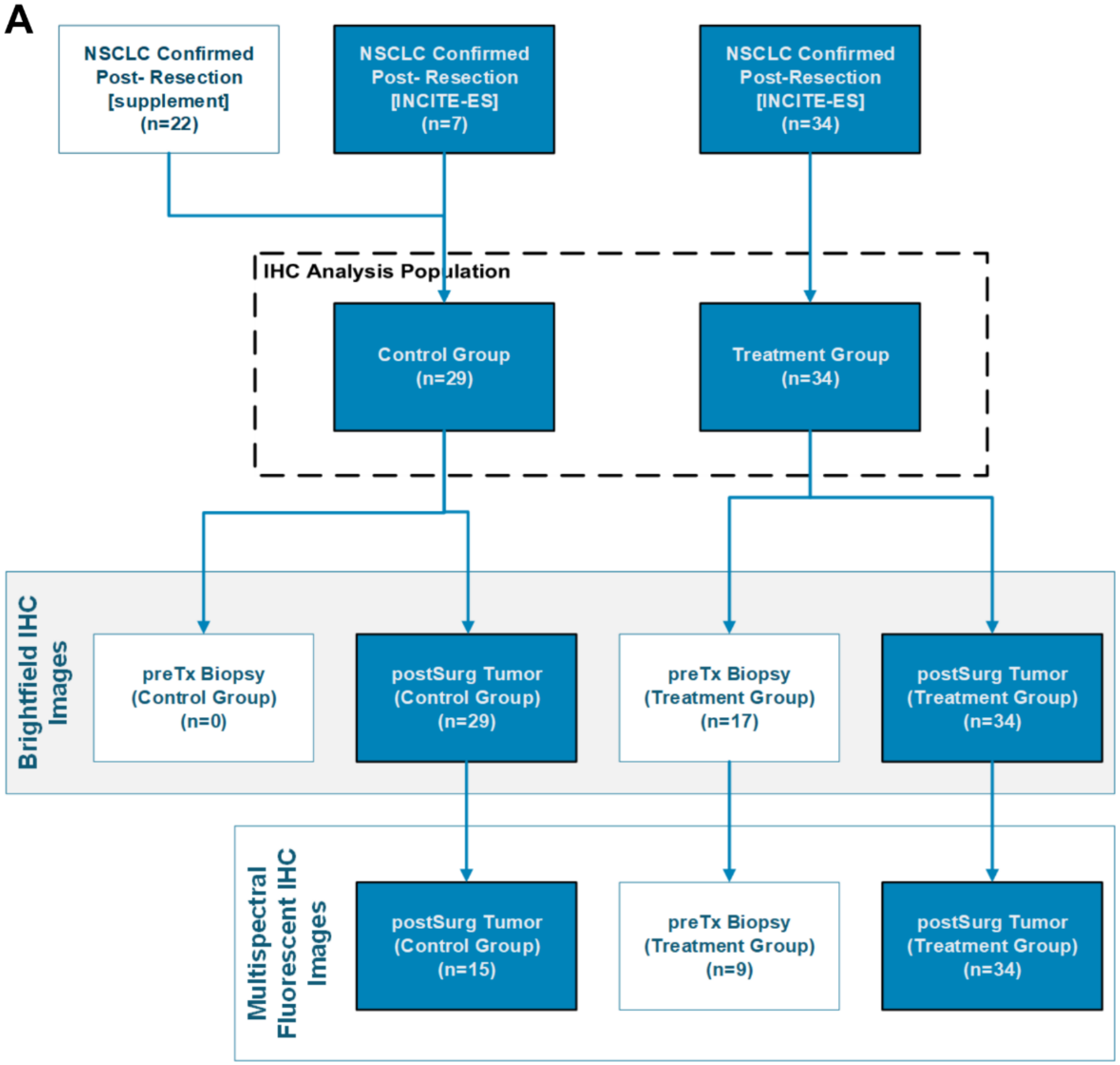

B **Brightfield IHC Lymphoid Structure Classification**

Feature	Immune Cell Aggregate	TLS	TLS_GC
B cells	≥50	≥50	≥50
T cells	<50	≥50	≥50
H&E GC	-	-	+

C **Fluorescent IHC Lymphoid Structure Classification**

Feature	Lymphoid Aggregate	TLS	TLS_GC
B cells	≥15	≥25	≥25
T cells	≥10	≥10	≥10
PNAd	0	≥1	≥1
$CD20^+$ AID^+ $Ki67^+$	-	≤13	>13

Supplementary Figure 3. Samples included in the brightfield IHC and multispectral fluorescent IHC analyses. (**A**) The INCITE ES PTE population, specifically the control group (n=7), was supplemented with additional, non-ablated resection samples from patients with characteristics similar to key eligibility criteria that were not enrolled in the study (n=22). This resulted in an IHC analysis population of n=29 control group, and a n=34 ablation group (including n=17 pre-ablation biopsy samples from ablation group patients with paired

samples). Multispectral IHC was conducted on a subset of these samples: 15 control samples representing the INCITE ES control patients and additional control samples, 9 pre-ablation biopsy samples, and all 34 post-ablation samples. (**B**) Brightfield IHC rubric used to assess the features that define each lymphoid structure. For this evaluation, TLS were defined as densely contiguous immune cell aggregates (ICA) of ≥ 50 $CD20^+$ B cells, and ≥ 50 T cells ($CD4^+$ or $CD8^+$), conspicuous at low power and typically nodular in configuration, and with an area of at least 14000 μm^2. The presence of a GC in each H&E image was classified as TLS_GC. (**C**) Multispectral IHC rubric used to assess the features that define each structure. Lymphoid aggregates (LA) were defined as structures containing at least 15 $CD20^+$ B cells, at least 10 $CD4^+$ T cells and zero $PNAd^+$ cells. TLS were defined as structures containing at least 25 $CD20^+$ B cells, at least 10 $CD4^+$ T cells, at least 1 $PNAd^+$ cell, and 13 or fewer $CD20^+$ AID^+ $Ki67^+$ triple-positive cells. TLS_GC met the TLS requirements and included a threshold of more than 13 $CD20^+$ AID^+ $Ki67^+$ triple-positive cells within the structure. (Created in BioRender. Pastori, C. (2026) https://BioRender.com/q0hrqb9).

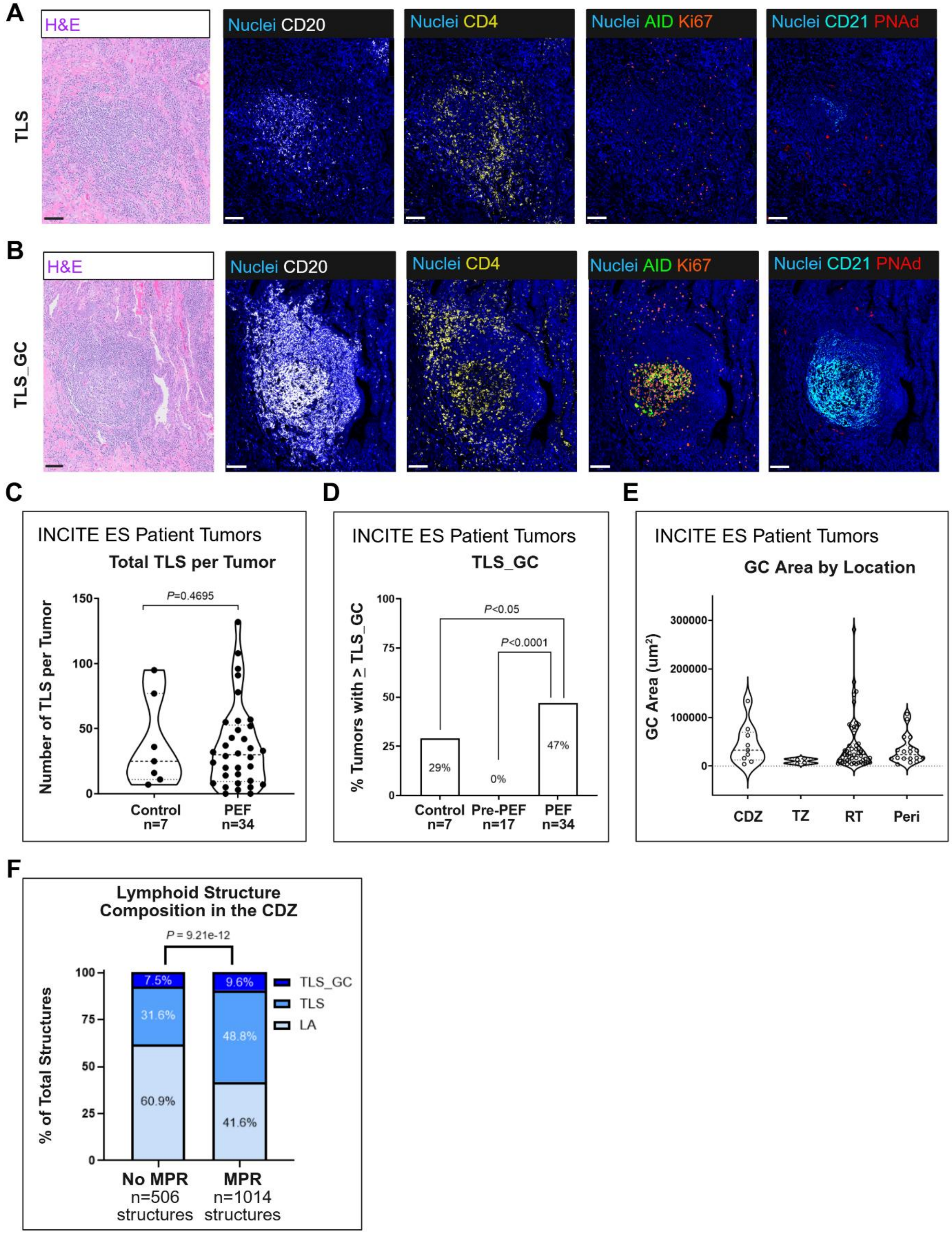

Supplementary Figure 4. Comparison of TLS and TLS_GC multispectral immunohistochemistry biomarkers, TLS quantification results from INCITE ES sample groups, and the association of TLS and GC formation with the pathological response to ablation. (**A-B**) Multispectral

immunofluorescent images from a representative TLS (**A**) and TLS_GC (**B**), with the associated H&E image shown for reference. All biomarkers are shown together (DAPI, CD20, CD4, AID, Ki67, CD21, PNAd), followed by CD20 alone to show B cells, CD4 alone to show T cells, AID and Ki67 together to show proliferating cells and class-switching recombination, and CD21 and PNAd to show follicular dendritic cell networks within the structure and proximal HEV. H&E images were captured at 20x magnification and fluorescent images captured at 10x magnification. Scale bars represent 100 µm. (**C**) Violin plot showing the total TLS counts by individual tumor for resection samples only from patients enrolled in the INCITE ES study (control group n=7, ablation group n=34) as assessed in the brightfield images. *P* values were calculated using the Mann Whitney test. (**D**) Bar plot demonstrating the percentage of tumors in the ablation group (n=34) with at least one TLS compared to pre-ablation biopsies (n=17) as well as to the non-ablated tumors from the control group (n=7) for resection samples only from patients enrolled in the INCITE ES study as assessed in the brightfield images ($P<0.0001$ among the three groups, with significant between-group differences as specified in the plot). *P* values were calculated using Fisher's exact test. (**E**) Violin plot demonstrating the GC area within the TLS in ablated tumors, indicating the GC in the CDZ are of similar size to those GC found in the remaining tumor (RT) and peritumoral area (Peri). No significant differences were observed between groups ($P=0.3123$ across the four groups) and *P* values were calculated using a one-way ANOVA after log transformation with Tukey's multiple comparisons test. (**F**) A statistically significant association between the types of lymphoid structure frequency (lymphoid aggregates [LA], TLS, TLS_GC) and major pathologic response (MPR) classification status was observed within the cellular depletion zone (CDZ) of ablation group tumors. The total number of lymphoid structures (LA, TLS, or TLS_GC) is shown for patients grouped by their CDZ MPR status. An independent pathologist reviewed the pathological response within the CDZ, which revealed an MPR (≤10% residual viable tumor) in 15 of 22 (68%) samples with an evaluable CDZ. To determine whether TLS

and GC formation differed between patients for which the CDZ reached MPR compared to patients for which the CDZ did not meet the MPR threshold, the distribution of the total number of lymphoid structures as defined by the multispectral fluorescent IHC was examined in each group (MPR versus no MPR). A Pearson's Chi-squared test was performed on the raw counts of each lymphoid structure type per patient group. The test revealed a statistically significant association between the types of lymphoid structure frequency and patient classification (MPR versus no MPR; $\chi^2(2) = 50.821$, $P<0.001$), indicating that the distribution of structures was not independent of MPR classification status. (Created in BioRender. Pastori, C. (2026) https://BioRender.com/bmi3s1l).

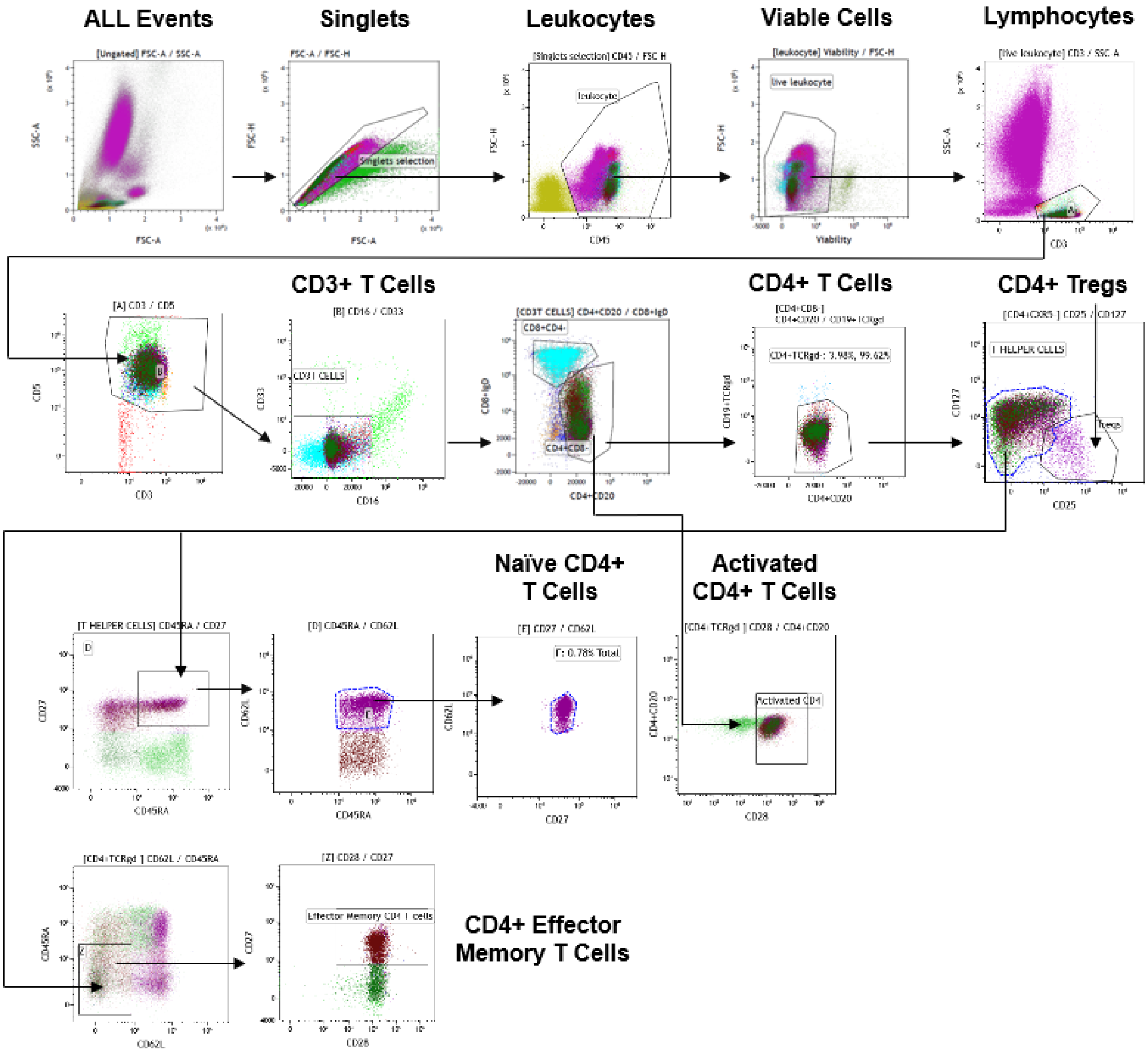

Population	Markers
CD4 T Cells	Defined as viable CD45+, CD3+, CD4+ T Cells
Naïve CD4 T Cells	Defined as CD27+, CD45RA+, CD62L+, CD4+ T Cells
Activated CD4 T Cells	Defined as CD28+, CD4+ T Cells
CD4 Effector Memory T Cells	Defined as CD45RA-, CD62L-, CD27+, CD28+, CD4+ T Cells
CD4 Treg Cells	Defined as CD25+, CD127-, CD4+ T cells

Supplementary Figure 5. Gating strategy for identification of $CD4^+$ T cell populations by flow cytometry. Flow cytometry gating was performed on live, singlet, $CD45^+$ leukocytes. $CD3^+$ T cells were further gated into $CD4^+$ subsets as listed in the table.

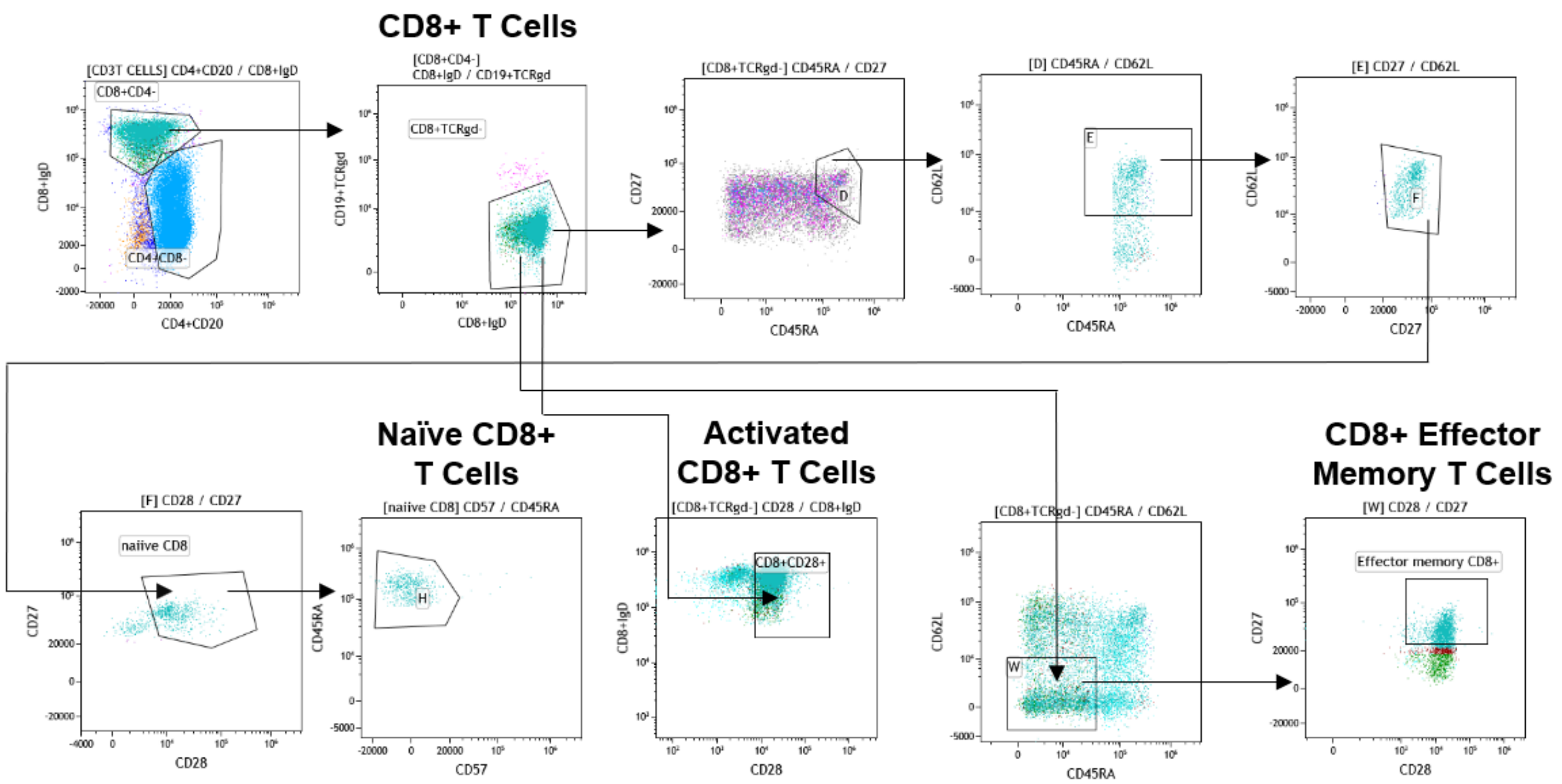

Population	Markers
CD8 T Cells	Defined as viable CD45+, CD3+, CD8+ T Cells
Naïve CD8 T Cells	Defined as CD27+, CD28+, CD45RA+, CD62L+, CD57-, CD8+ T Cells
Activated CD8 T Cells	Defined as CD28+, CD8+T Cells
CD8 Effector Memory T Cells	Defined as CD45RA-, CD62L-, CD27+, CD28+, CD8+ T Cells

Supplementary Figure 6. Gating strategy for identification of $CD8^+$ T cell populations by flow cytometry. Flow cytometry gating was performed on live, singlet, $CD45^+$ leukocytes. $CD3^+$ T cells as shown in Figure S5 were further gated into $CD8^+$ subsets as listed in the table.

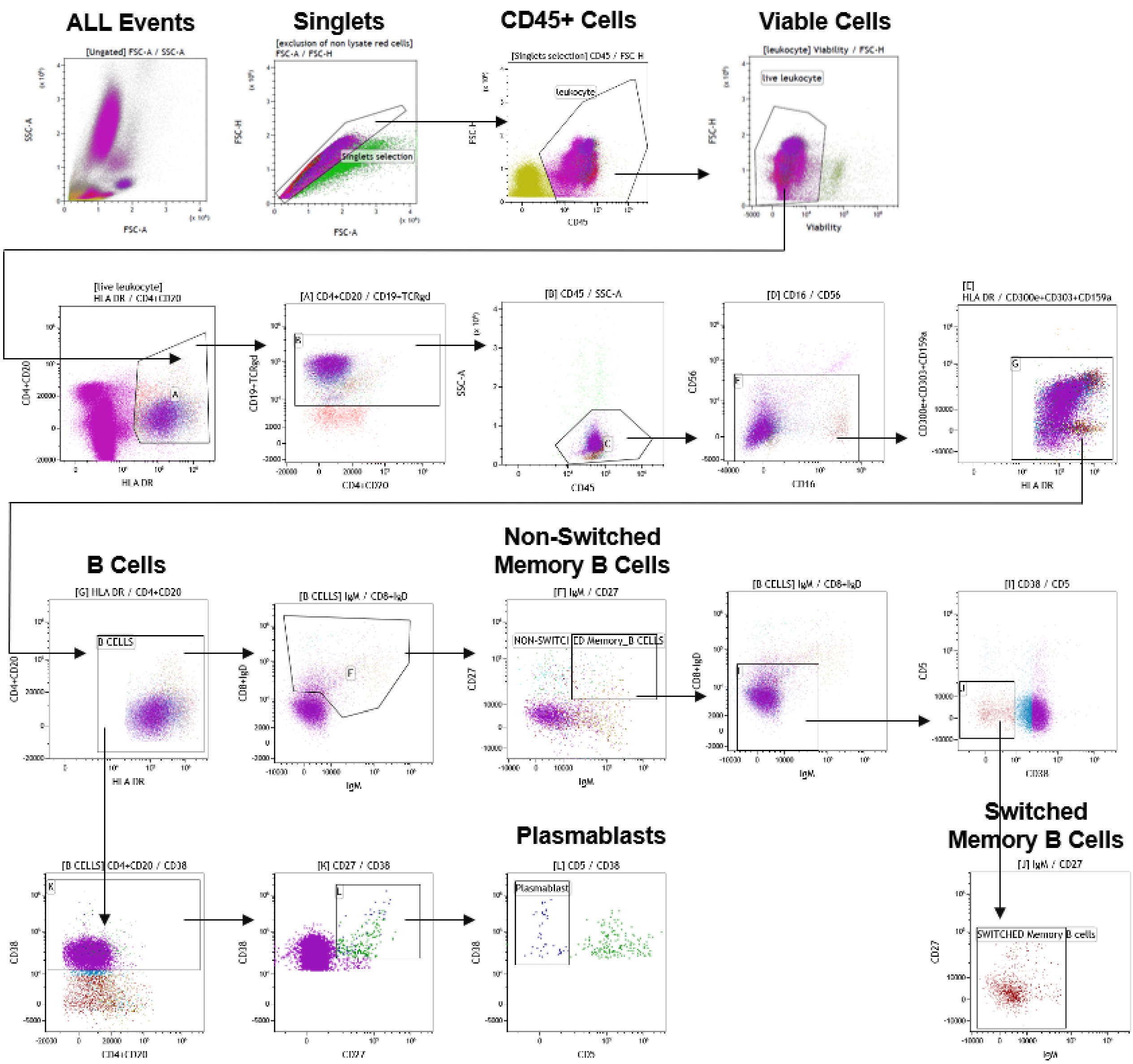

Population	Markers
B Cells	Defined as viable CD45+, CD19+, CD20+ Cells
Non-Switched Memory B Cells	Defined as CD27+, IgM+, IgD+, CD5-, CD38- B Cells
Switched Memory B Cells	Defined as IgM-, IgD-, CD5-, CD38-, CD27-/+ B Cells
Plasmablasts	Defined as CD19+, CD20-, CD38++ B Cells

Supplementary Figure 7. Gating strategy for identification of B cell populations by flow cytometry. Flow cytometry gating was performed on live, singlet, $CD45^+$ leukocytes. B cell subsets were identified as listed in the table.

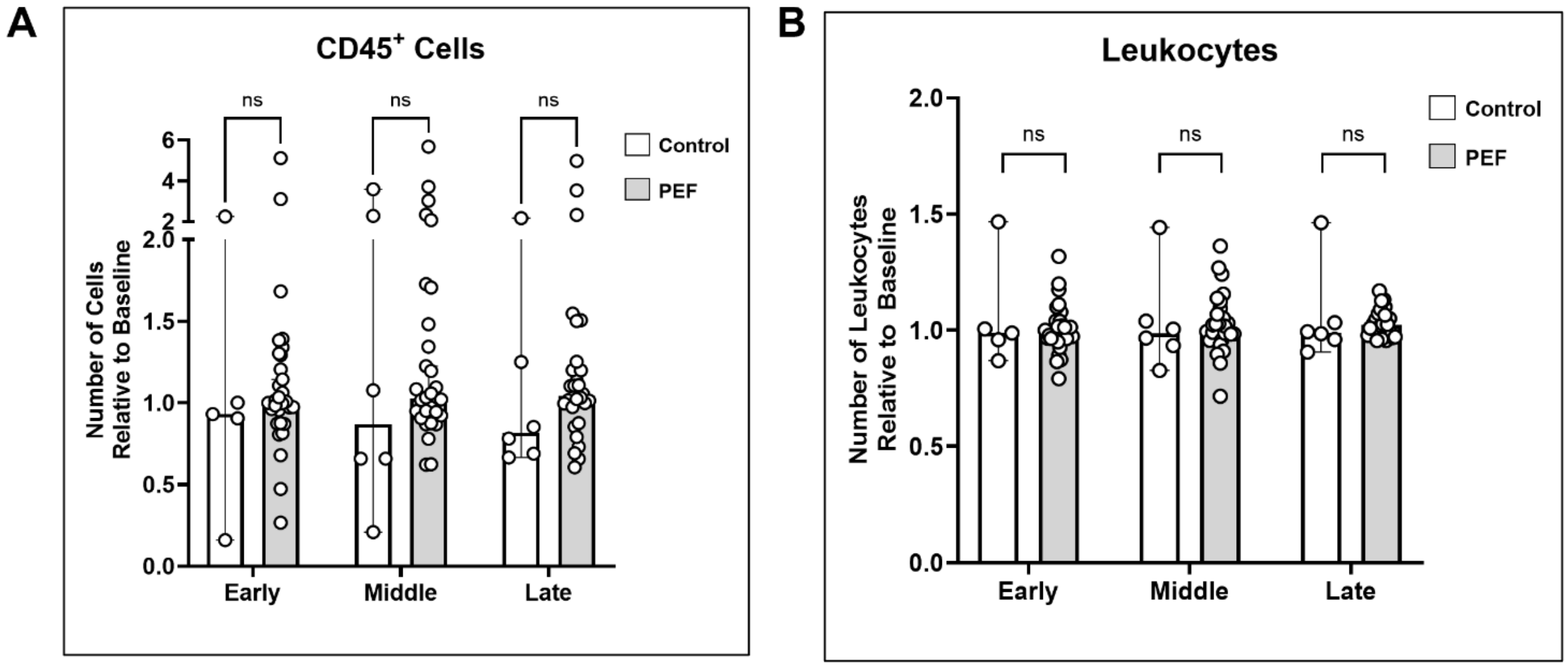

Supplementary Figure 8. Analysis of peripheral blood samples by flow cytometry. Bar plots demonstrating the total numbers of $CD45^+$ cells (**A**) and the total number of leukocytes (**B**) were not significantly different before or after ablation or between samples from the control and ablation groups at each time point collected. The mean percent of cells relative to the day 0 biopsy (pre-ablation in the ablation group) is shown (± SD) from available patient samples (control group n=6, ablation group n=26) that were collected 15-30 days post-biopsy/ablation. *P* values were determined by Wilcoxon rank-sum test.

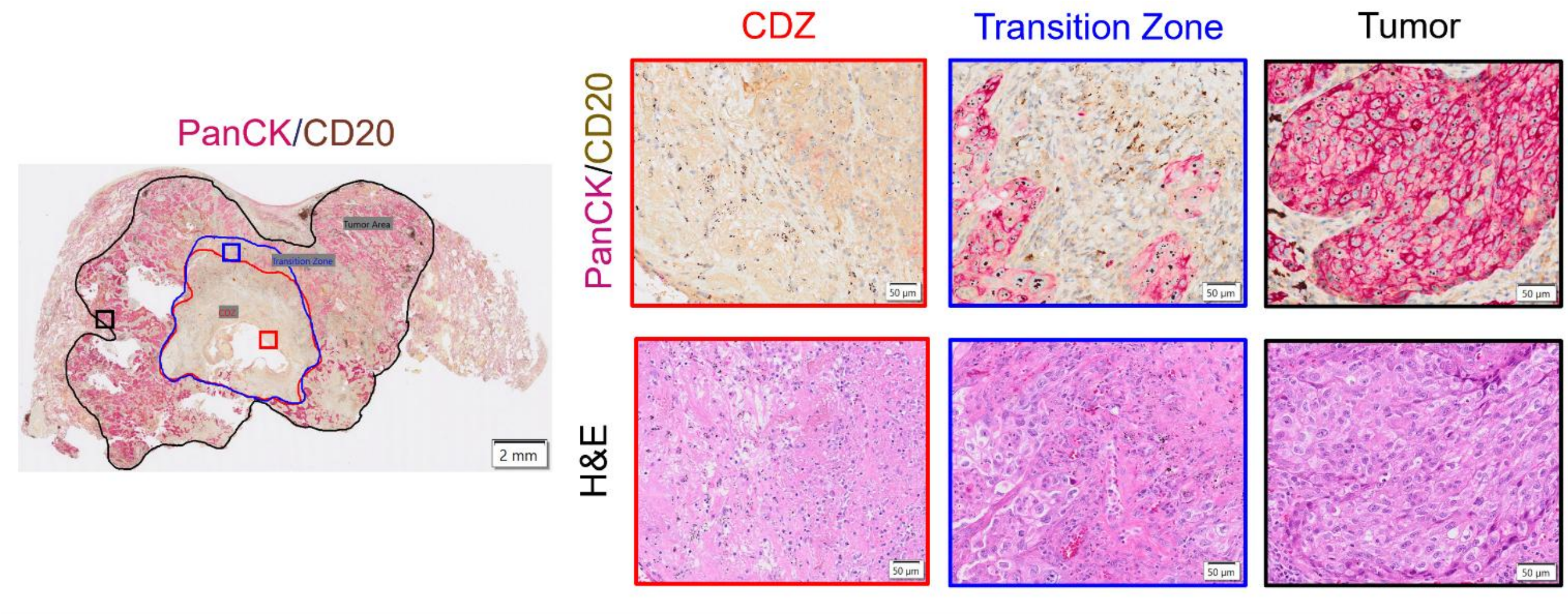

Supplementary Figure 9. Histopathological example of the ablation-induced cellular depletion zone (CDZ). Representative example of an ablated tumor sectioned perpendicular to the ablation needle placement path using the CT scans as a reference for the angle of needle placement. Gaps in the tissue correspond to punch biopsies collected for evaluation of gene expression and tissue cytokines. Scale bar represents 2 mm. Serial tissue sections were stained with PanCK/CD20 dual stain (top panel) or H&E (bottom panel), and images were annotated to indicate the tumor (black outline), transition zone (blue outline) and CDZ (red outline). PanCK (dense pink) stains epithelial-derived cancer cells (carcinomas). Although non-tumor cells of epithelial origin also stain for panCK, the cancerous and non-cancerous cells can be distinguished by their morphology. CD20 (brown) identifies B cells. Higher magnification images from the regions denoted by the colored squares in the whole-tissue image are shown to the right. Scale bars represent 50 µm. The CDZ region is characterized as being devoid or nearly devoid of viable cancerous cells, visualized by the pale hue remaining and the absence of PanCK^{+} tumor cells, whereas the transition zone demonstrated a greater density of tumor cells than the CDZ but less than that seen in the remaining non-ablated tumor.

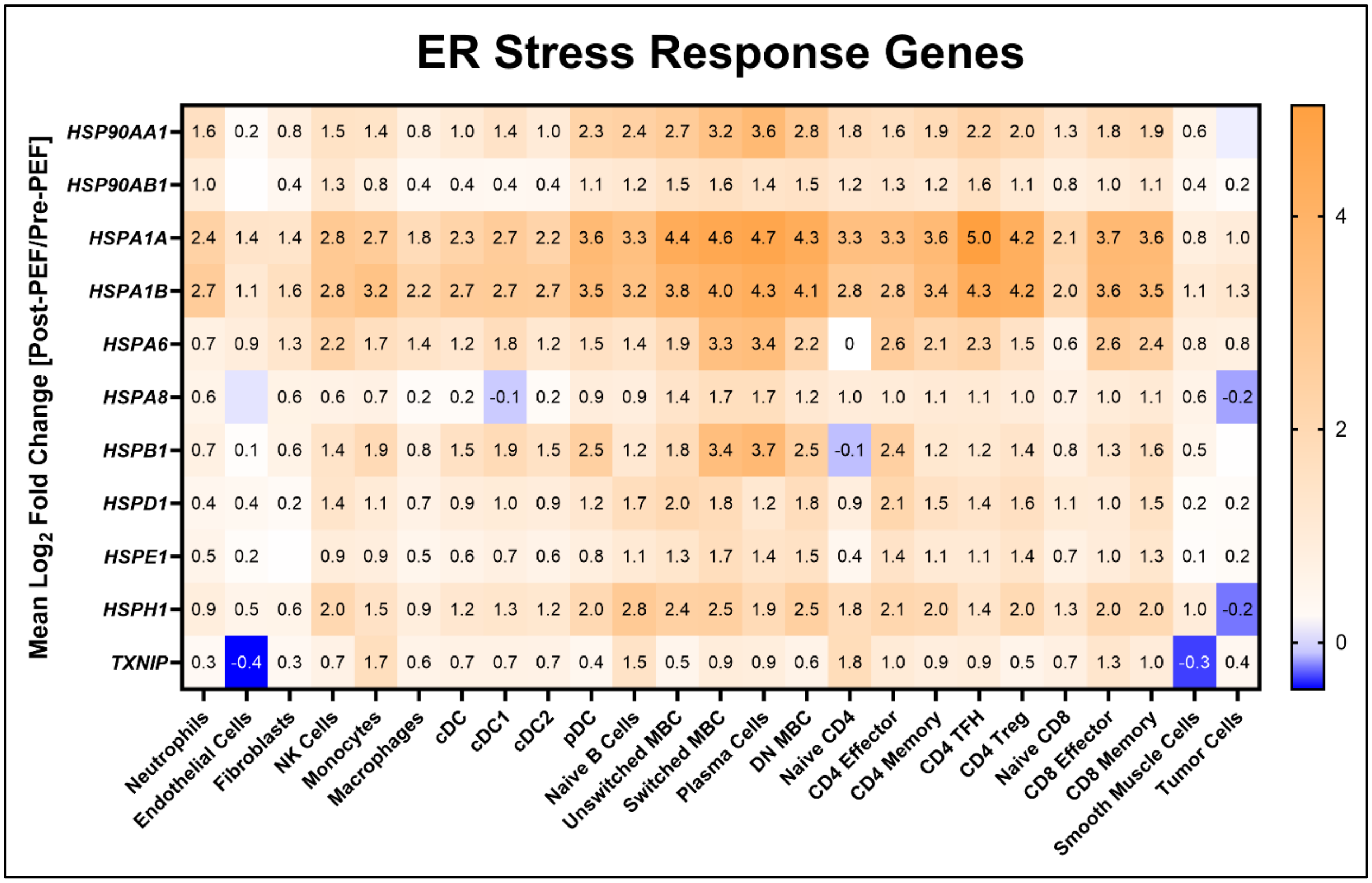

Supplementary Figure 10. Ablation induces increased expression of stress response genes. Heatmap showing the mean $\log_2$ fold change in expression of endoplasmic reticulum (ER) stress response genes in resected tumor tissue from patients that received ablation (n=25) relative to pre-ablation biopsies (n=20) analyzed by scRNA-seq.

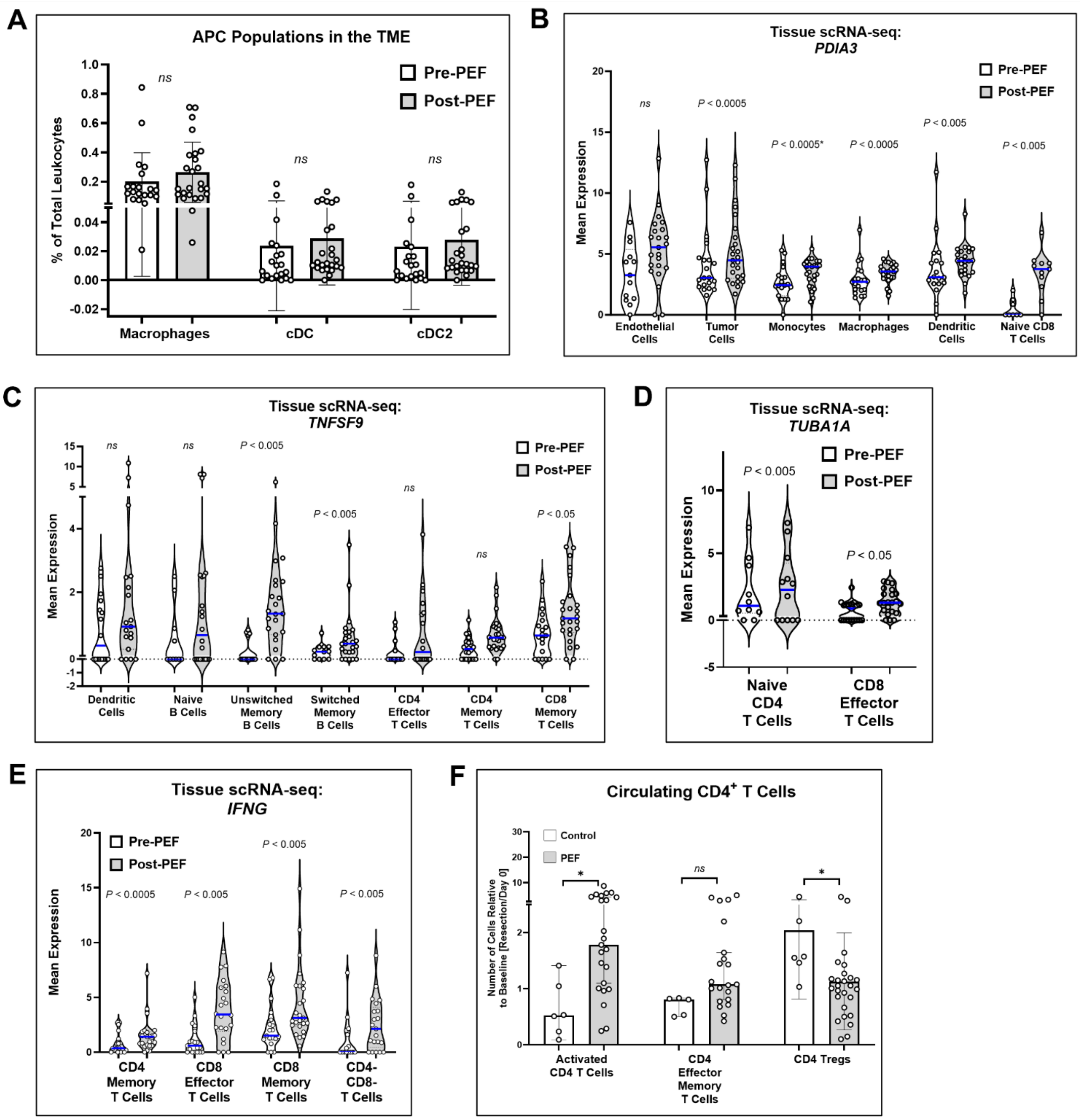

Supplementary Figure 11. Ablation induces increased expression of genes involved in antigen recognition and T lymphocyte activation as well as increased activated and memory T lymphocytes in circulation. (**A**) Increases in antigen presenting cell (APC) populations were not significant in the TME by the time of resection post-ablation as assessed by scRNA-seq (pre-ablation n=20, post-ablation n=25). Bar plots represent the mean percent of each cell population shown relative to the total number of leukocytes in each sample (± SD). *P* values were calculated using the propeller

method. (**B-E**) Gene expression levels analyzed by scRNA-seq are shown across multiple cell types for the genes specified (pre-ablation n=20, post-ablation n=25). Blue lines represent the median, and *P* values were calculated using the Wilcoxon rank-sum test. Asterisks (*) indicate samples for which the adjusted *P* value was also significant ($P<0.05$). Expression changes that were not significant are indicated by (ns). This analysis revealed significant increases in genes involved in the presentation of antigens to T cells and their subsequent activation (**B-C**), as well as genes involved in T cell cytoskeleton reorganization linked to the immune synapse formation (**D**). Interferon gamma was also elevated in cytotoxic and helper T cells, which can result from T cells being activated by antigens (**E**). (**F**) Flow cytometry analysis of peripheral blood lymphocytes also revealed a significant increase in activated $CD4^+$ T cells, a trend towards increased $CD4^+$ effector memory T cells, and a significant decrease in $CD4^+$ T regulatory (T_{reg}) cells post-ablation. The mean number of cells relative to the day 0 biopsy (pre-ablation in the ablation group) is shown (± SD) from available patient samples (control group n=6, ablation group n=26) that were collected 15-30 days post-biopsy/ablation. *P* values were calculated using the Wilcoxon rank-sum test.

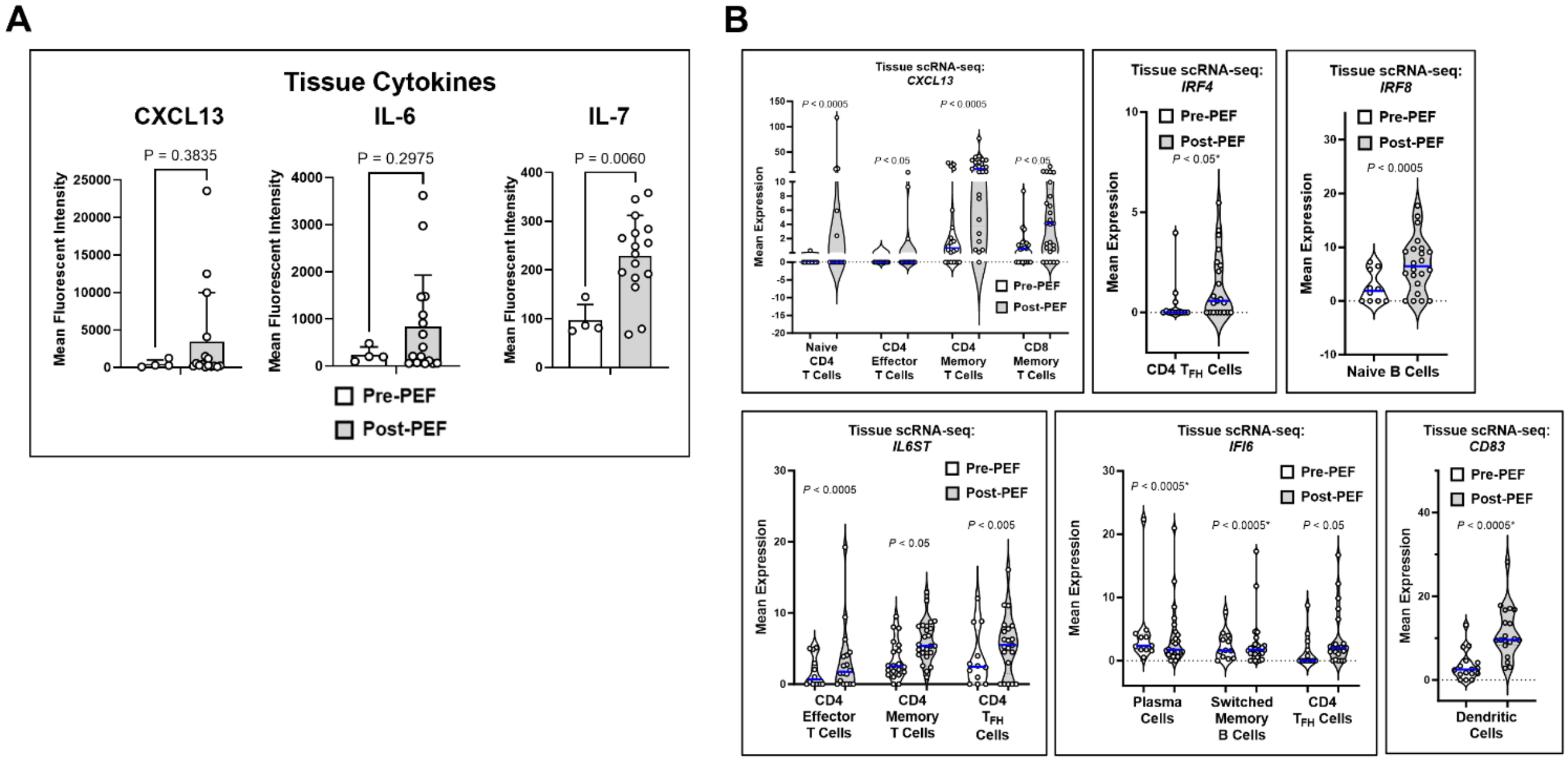

Supplementary Figure 12. Genes critical for GC formation and activity are significantly elevated post-ablation. (**A**) Bar plots represent the mean FI (± SD) for the indicated cytokines pre-ablation (n=4) and post-ablation (n=16). *P* values were generated using a two-tailed, unpaired t-test. CXCL13 is central to B and T cell recruitment and organization within lymphoid structures and B cell development and survival ([1]). IL-6 promotes differentiation of $CD4^+$ T follicular helper (T_{FH}) cells, which promote GC formation, and induces B cell activation and plasma cell survival and antibody secretion ([2]). IL-7 plays multiple roles during TLS development and GC formation, including promotion of HEV establishment, B cell survival, proliferation, and maturation, and promoting the survival of memory T cells ([3,4]). (**B**) Violin plots representing the mean expression of genes critical for GC formation and the interaction and differentiation of the cell types within as measured by scRNA-seq. Mean expression levels by cell population are shown in pre-ablation (n=20) compared to post-ablation (n=25). Blue lines represent the median, and *P* values were calculated using the Wilcoxon rank-sum test. Asterisks (*) indicate samples for which the adjusted *P* value was also significant ($P<0.05$). *CXCL13* expression was significantly increased in multiple T cell populations. Additional changes included increased *IRF4*, which can be expressed in response to T cell receptor stimulation

and is also a key transcriptional regulator in B cells ([5]); increased *IRF8*, involved in modulating the GC reaction and linked to B cell survival ([6]); increased *IL6ST*, part of the IL-6 receptor complex that drives T cell differentiation towards T_{FH} formation and promotes GC formation ([2]); increased *IFI6*, whose expression in DCs augments the generation of T_{FH} cells while expression in B and T cells is linked to type I interferon-dependent enhancement of humoral immunity ([7]), and increased *CD83*, which is upregulated by interferon response factors and promotes DC maturation.

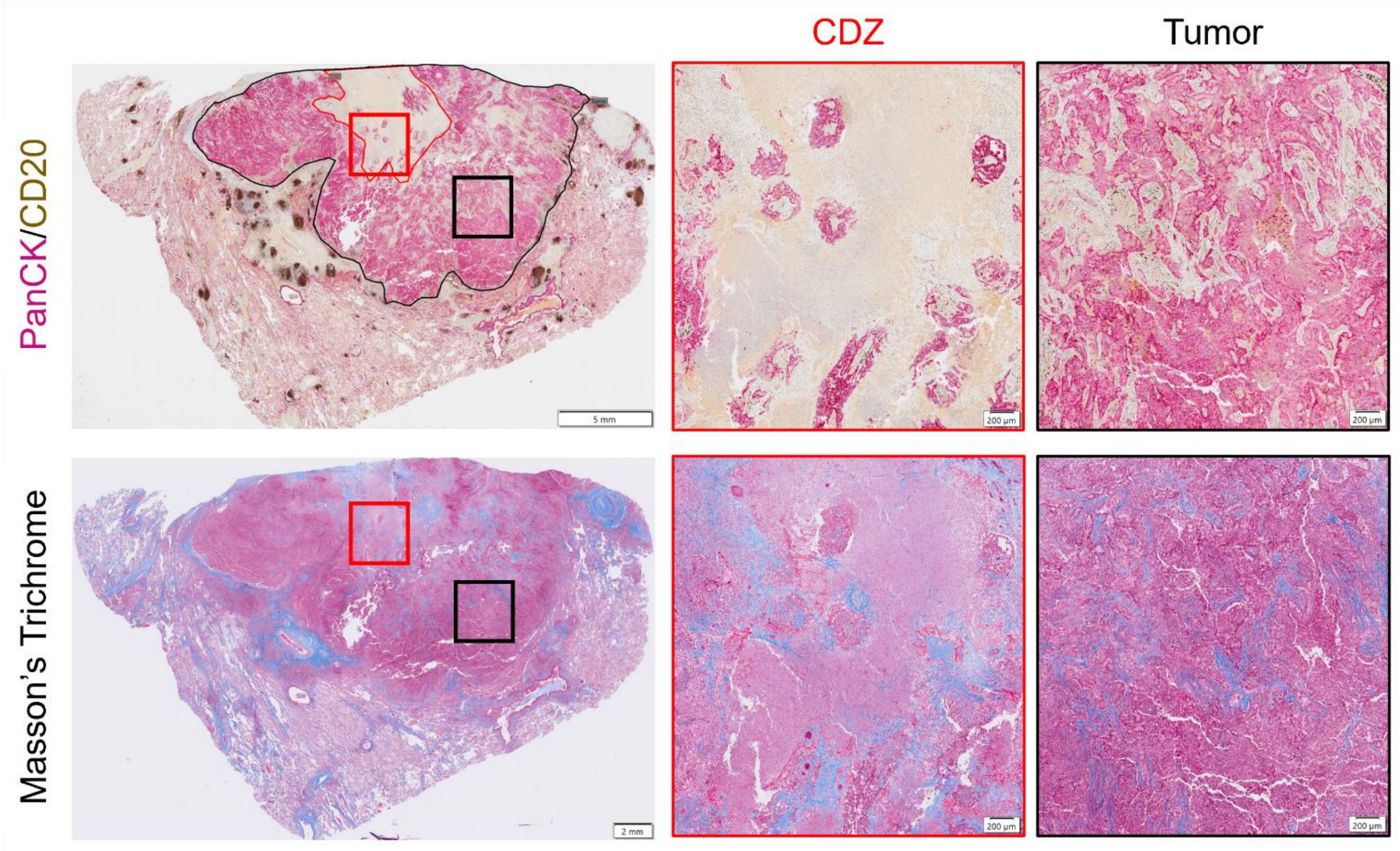

Supplementary Figure 13. Ablation does not result in encapsulation around the CDZ. Representative images of the CDZ (red outline) and remaining tumor (black outline) in an ablated tumor tissue section stained with PanCK/CD20 dual stain (top panel). Staining of a serial section with Masson's trichrome (blue) (bottom panel) revealed a lack of encapsulation around the CDZ, with no qualitative differences in the connective tissue/collagen fiber between the CDZ and remaining tumor. Higher magnification images from the regions denoted by the colored squares in the whole-tissue image are shown to the right.

A

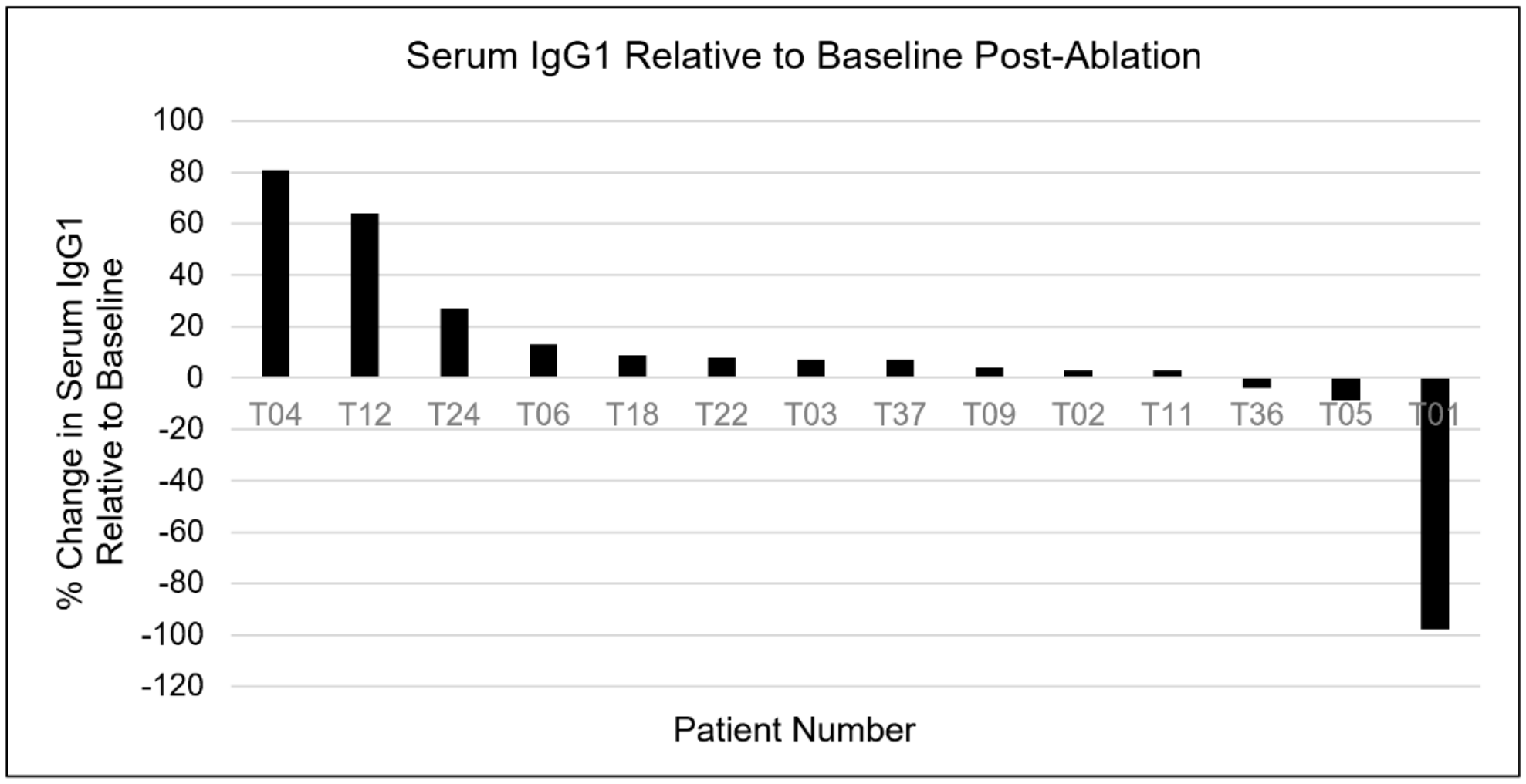

B

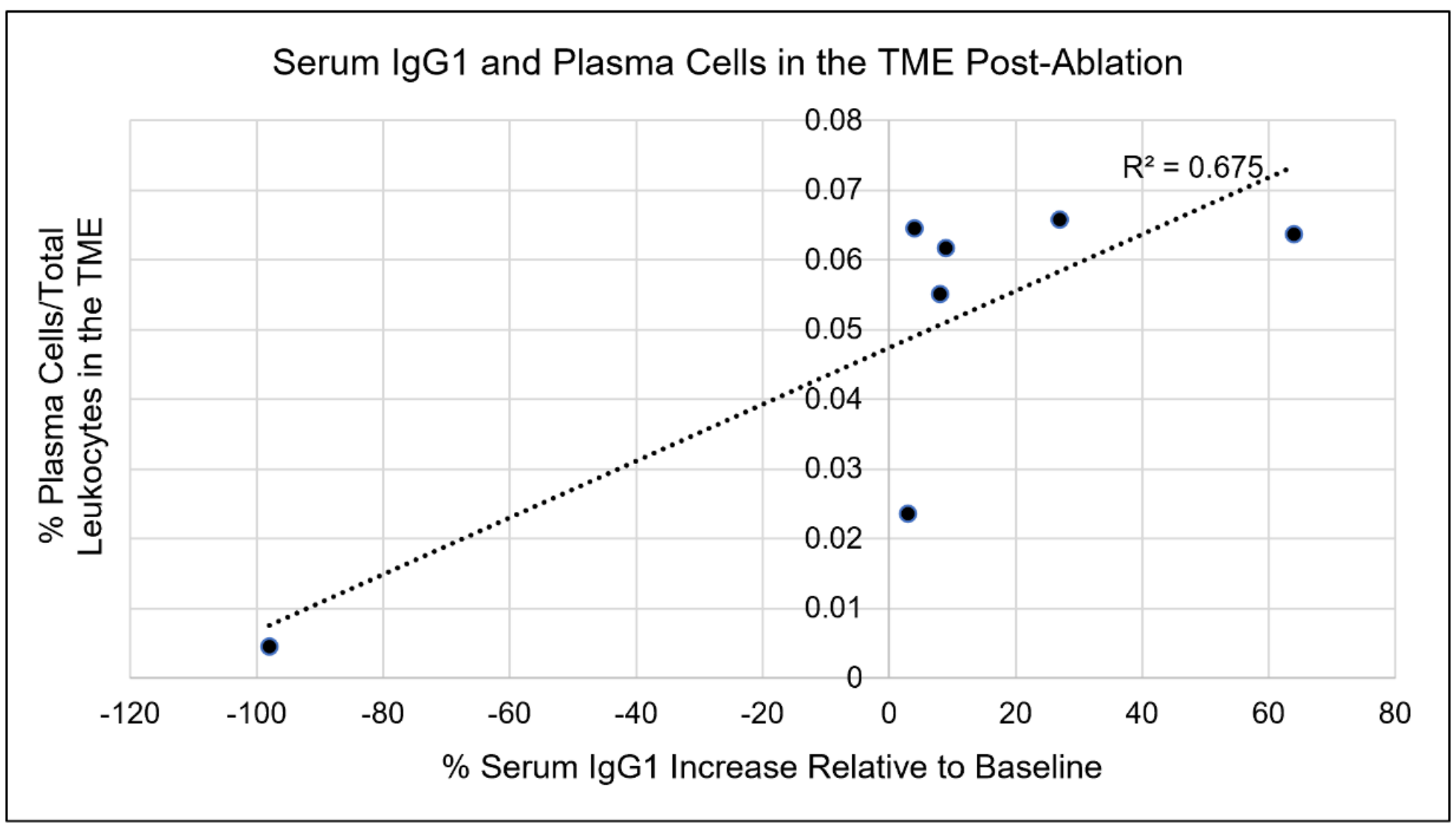

C

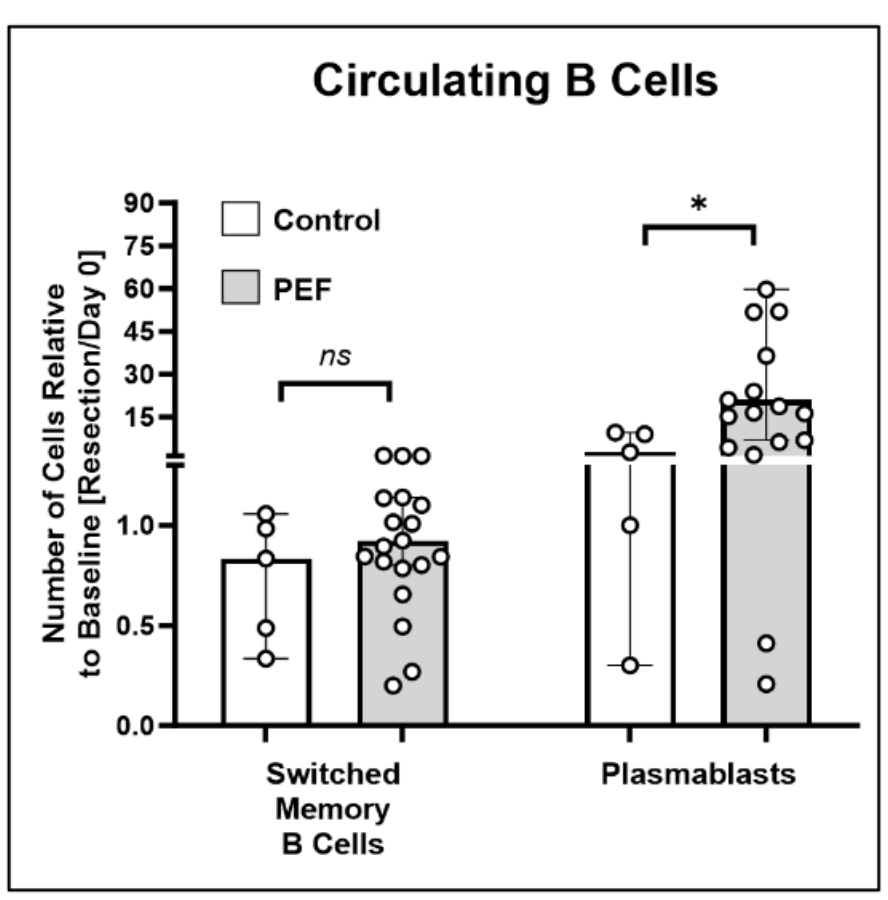

Supplementary Figure 14. Ablation induces a humoral immune response that results in increased serum IgG1, switched memory B cells, and plasmablasts in circulation. (A) Analysis of

serum immunoglobulin G (IgG) demonstrated elevated biopsy-specific IgG levels in 11/14 patients with available remaining samples. Serum collected prior to the resection procedure was compared to serum collected prior to the ablation procedure (baseline) via an ELISA assay using plates coated with tumor lysate collected from the pre-ablation biopsy. Serum IgG was elevated by more than 25% in 3 patients, and all 3 had TLS_GC in the resected tumor sample evaluated as confirmed by the multispectral GC panel immunostaining. (**B**) Analysis of the percent of plasma cells (relative to total leukocytes) in the tumor microenvironment (TME) as assessed by scRNA-seq indicated a positive correlation (R2=0.675, $P<0.05$) in the percent of plasma cells present post-ablation and the percent increase in serum IgG levels in the patients that had both serum IgG and scRNA-seq data available for the analysis (n=7). *P* values were calculated using a Pearson correlation. (**C**) Flow cytometry analysis of peripheral blood lymphocytes revealed significant increases in plasmablasts and a trend towards increased switched memory B cells post-ablation. The mean percent of cells relative to the day 0 biopsy (pre-ablation in the ablation group) is shown (± SD) from available patient samples (control group n=6, ablation group n=26) that were collected 15-30 days post-biopsy/ablation. *P* values were calculated using the Wilcoxon rank-sum test.

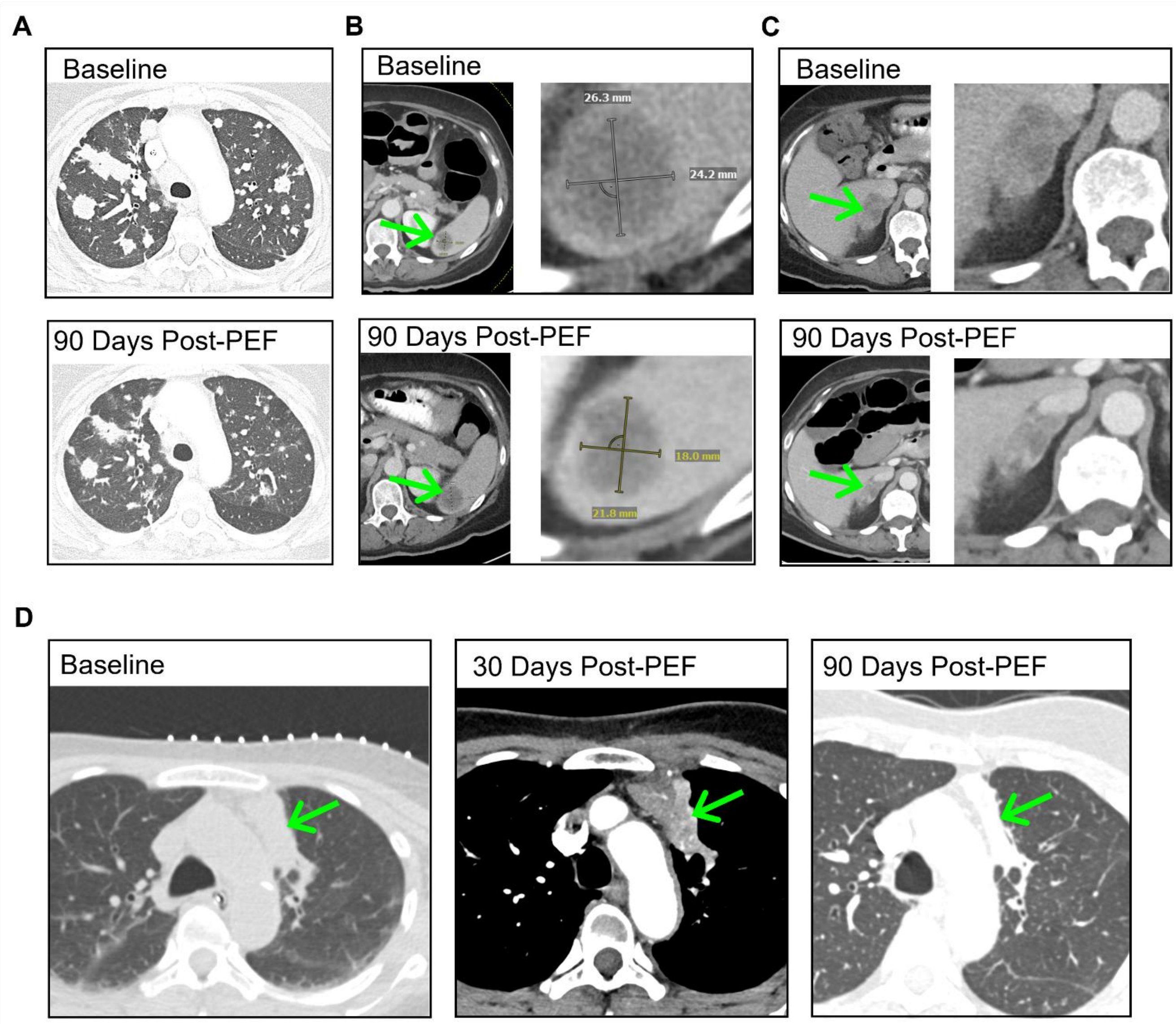

Supplementary Figure 15. Off-target responses observed in non-ablated lesions distant from those ablated with the Aliya System in patients not part of the INCITE ES study. (**A-C**) Pre-ablation and post-ablation CT scans from a first case in a patient with stage IV colon cancer with diffuse metastatic disease impacting multiple organs including lung, spleen, and liver that had progressed through multiple prior treatments, including pembrolizumab. Two among many metastatic lesions within the lung (**A**) were ablated. These lesions were completely ablated, and the energy was delivered for palliative purposes in the setting of FOLFIRI (folinic acid, fluorouracil, and irinotecan) and panitumumab (monoclonal antibody inhibitor of EGFR). By 90 days post-ablation, notable

decreases in lesion size (green arrows) were noted in the spleen **(B)** and liver **(C)**, despite those lesions not having been ablated. (**D**) Pre-ablation and post-ablation CT scans from a second case in a patient with stage IV metastatic lung carcinoma that had an upper lung nodule ablated palliatively in the setting of first line pembrolizumab (monoclonal antibody inhibitor of PD-1), pemetrexed (antineoplastic agent), and carboplatin (platinum chemotherapy) followed by maintenance with pembrolizumab and pemetrexed. By 90 days post-ablation, the adrenal metastasis was gone and a pleural lesion (green arrow) resolved, neither of which had received the specialized energy.

References

1. Sautès-Fridman C, Petitprez F, Calderaro J, Fridman WH. Tertiary lymphoid structures in the era of cancer immunotherapy. *Nat Rev Cancer*. **2019**;19(6):307-325. doi:10.1038/s41568-019-0144-6.

2. Elsner RA, Shlomchik MJ. Germinal Center and Extrafollicular B Cell Responses in Vaccination, Immunity, and Autoimmunity. *Immunity*. **2020**;53(6):1136-1150. doi:10.1016/j.immuni.2020.11.006.

3. Ware MB, Wolfarth AA, Goon JB, et al. The Role of Interleukin-7 in the Formation of Tertiary Lymphoid Structures and Their Prognostic Value in Gastrointestinal Cancers. *J Immunother Precis Oncol*. **2022**;5(4):105-117. doi:10.36401/JIPO-22-10.

4. Knop L, Deiser K, Bank U, et al. IL-7 derived from lymph node fibroblastic reticular cells is dispensable for naive T cell homeostasis but crucial for central memory T cell survival. *Eur J Immunol*. **2020**;50(6):846-857. doi:10.1002/eji.201948368.

5. Sciammas R, Shaffer AL, Schatz JH, Zhao H, Staudt LM, Singh H. Graded Expression of Interferon Regulatory Factor-4 Coordinates Isotype Switching with Plasma Cell Differentiation. *Immunity*. **2006**;25(2):225-236. doi:10.1016/j.immuni.2006.07.009.

6. Lu R. Interferon regulatory factor 4 and 8 in B-cell development. *Trends Immunol*. **2008**;29(10):487-492. doi:10.1016/j.it.2008.07.006.

7. Dahlgren MW, Plumb AW, Niss K, Lahl K, Brunak S, Johansson-Lindbom B. Type I Interferons Promote Germinal Centers Through B Cell Intrinsic Signaling and Dendritic Cell Dependent Th1 and Tfh Cell Lineages. *Front Immunol*. **2022**;13:932388. doi:10.3389/fimmu.2022.932388.
