# Supplementary material for: Pulsed Electric Field Ablation Reprograms Tumor Immunity and Stimulates Germinal Center Formation in Tertiary Lymphoid Structures in Patients with Non–Small Cell Lung Cancer
Source: Clin Cancer Res. 2026 Jun 17;32(17):3830–48. doi: 10.1158/1078-0432.CCR-25-3847 (PMC13530995; doi:10.1158/1078-0432.CCR-25-3847)
Supplement: Supplementary file 2 [file ccr-25-3847_supplementary_table_s1_suppts1.docx]

| **Supplementary Table 1. Representativeness of Study Participants** | |
| --- | --- |
| Cancer Type | Resectable non-small cell lung carcinoma (NSCLC) IA2-IB. |
| Sex | Significantly more males (73.4%) are diagnosed with NSCLC in Spain than females (26.6%). Early-stage NSCLC is slightly more prevalent in males (52%) in the Netherlands. NSCLC incidence rates are slightly higher in males, but early-stage NSCLC is more prevalent in women in Hong Kong. Early-stage NSCLC is slightly more prevalent in males than females in patients from the United States, regardless of race or ethnicity. |
| Age | The median age at diagnosis for patients with NSCLC is approximately 67-70 years worldwide, 71 years in Stage II NSCLC patients in the Netherlands, and 66 years for early-stage NSCLC in Spain. The median age at diagnosis is 70 years for early-stage NSCLC patients in the United States, though a subset of patients are diagnosed before age 40. |
| Race/ethnicity | White/Caucasian individuals tend to have the highest incidence of NSCLC. Black patients may be diagnosed with NSCLC at a younger age but have a lower percentage of local-stage diagnoses. Asian/Pacific Islander patients may have a higher percentage of distant-stage diagnoses. |
| Geography | It is estimated that more than one of every three lung cancers occurs in China. Lung cancer is the second most frequent cancer in the United States, and the number one cause of cancer deaths for both sexes worldwide. NSCLC accounts for 80-85% of all lung cancer cases in Western countries, and 94% of all lung cancers in Hong Kong. |
| Other considerations | Women continue to be underrepresented in NSCLC clinical trials. Black and Hispanic patients remain underrepresented in clinical trials for breast, colorectal, lung, and prostate cancer. |
| Overall representativeness of this study | The age distribution of our study population is similar to the age of initial diagnosis in most early-stage NSCLC patients in the literature. The majority of study patients were enrolled at centers in Spain, so the greater prevalence of male patients in our study (68%) is reflective of the higher rate of NSCLC diagnoses in men in Spain. Similarly, the race and ethnicity demographics of our study population (95% white/Caucasian, 86% Hispanic or Latino) reflect the demographics from where they enrolled. |
