# Supplementary material for: Pulsed Electric Field Ablation Reprograms Tumor Immunity and Stimulates Germinal Center Formation in Tertiary Lymphoid Structures in Patients with Non–Small Cell Lung Cancer
Source: Clin Cancer Res. 2026 Jun 17;32(17):3830–48. doi: 10.1158/1078-0432.CCR-25-3847 (PMC13530995; doi:10.1158/1078-0432.CCR-25-3847)
Supplement: Supplementary file 3 [file ccr-25-3847_supplementary_table_s2_suppts2.docx]

| **Supplementary Table 2. Multispectral Immunofluorescent Antibodies** | | | | | | | |
| --- | --- | --- | --- | --- | --- | --- | --- |
| **Manufacturer** | **Product Number** | **Target** | **Clone** | **AR** | **Block** | **Secondary HRP** | **Opal** |
| Sigma | MABF2050 | Peripheral Node Addressin (PNAd) | MECA-79 | 9 | Biocare Ren^2^ | Akoya | 690 |
| Novus | NBP1-43375 | Mouse-anti-rat IgM^1^ | HIS40 | N/A | Biocare Ren^2^ | N/A | N/A |
| Biocare | API3209AA | CD4 | EP204 | 9 | Akoya | Akoya | 570 |
| Abcam | ab269454 | AID (Anti-AICDA) | EPR23436-45 | 9 | Akoya | Akoya | 520 |
| Leica | NCL-L-CD21-2G9 | CD21 | 2G9 | 6 | Akoya | Akoya | 480 |
| Epredia | RM9106S | Ki67 | SP6 | 6 | Akoya | Akoya | 620 |
| Leica | NC-L-CD20-L26 | CD20 | L26 | 6 | Akoya | Akoya | 780 |
| ^1^ *Secondary antibody for PNAd primary (rat) antibody*  ^2^ *Biocare Renaissance diluent* | | | | | | | |
