# Supplementary material for: Pulsed Electric Field Ablation Reprograms Tumor Immunity and Stimulates Germinal Center Formation in Tertiary Lymphoid Structures in Patients with Non–Small Cell Lung Cancer
Source: Clin Cancer Res. 2026 Jun 17;32(17):3830–48. doi: 10.1158/1078-0432.CCR-25-3847 (PMC13530995; doi:10.1158/1078-0432.CCR-25-3847)
Supplement: Supplementary file 4 [file ccr-25-3847_supplementary_table_s3_suppts3.docx]

| **Supplementary Table 3. Single-Cell RNA Sequencing Cell Classification Scheme** | |
| --- | --- |
| **Population Name** | **Identification Criteria Notes** |
| **LEUKOCYTES** | **Leukocytes defined as: *PTPRC+,* CD3+ or CD19+ or CD16+ or CD14+, *EPCAM-*** |
|  |  |
| **B Cells** | **Defined as CD19+ CD3- CD16- CD4- CD8- HLA-DR+ *CD79A+*** |
| Naïve B Cell | Defined within B cells as CD27- IgM+ IgD+ |
| Double Negative Memory B Cell | Defined within B cells as CD27- IgD- |
| Switched Memory B Cell (MBC) | Defined within B cells as CD27+ IgM- |
| Switched MBC (IgA) | Defined within switched memory B cells, as *IGHA1^hi^* or *IGHA2^hi^* |
| Switched MBC (IgD) | Defined within switched memory B cells, as *IGHD^hi^* |
| Switched MBC (IgE) | Defined within switched memory B cells, as *IGHE^hi^* |
| Switched MBC (IgG) | Defined within switched memory B cells, as *IGHG1^hi^ or IGHG2^hi^ or IGHG3^hi^ or IGHG4^hi^* |
| Unswitched MBC | Defined within B cells as CD27+ IgM+ and IgD+ |
| Plasma Cell | Defined within B cells as CD27+, *CD38^hi^, SDC^hi^* and/or *SLAMF7^hi^, CD24-* |
| Plasma Cell (IgA) | Defined within plasma cells, as *IGHA1^hi^* or *IGHA2^hi^* |
| Plasma Cell (IgD) | Defined within plasma cells, as *IGHD^hi^* |
| Plasma Cell (IgE) | Defined within plasma cells, as *IGHE^hi^* |
| Plasma Cell (IgG) | Defined within plasma cells, as *IGHG1^hi^ or IGHG2^hi^ or IGHG3^hi^ or IGHG4^hi^* |
| Plasma Cell (IgM) | Defined within plasma cells, as *IGHM^hi^* |
|  |  |
| **T Cells** | **Defined as CD3+, CD4+ and/or CD8+, CD19- CD16- CD14-** |
| Double Negative  T Cell | Defined within T Cells as CD4-, CD8- |
| Double Positive  T Cell | Defined within T Cells as CD4+, CD8+ |
| **CD4+ T Cells** | **Defined within T Cells as CD4+, CD8-** |
| Naïve CD4 T Cell | Defined within CD4+ T cells as: CD27+, CD45RA+, CD62L+ |
| Central Memory  CD4 T Cell | Defined within CD4+ T cells as: CD27+, CD45RA-/dim, CD62L+ |
| Transitional Memory  CD4 T Cell | Defined within CD4+ T cells as: CD27+, CD45RA, CD62L- |
| Peripheral Memory  CD4 T Cell | Defined within CD4+ T cells as: CD27-, CD45RA-, CD62L-/+ |
| Terminal Effector  CD4 T Cell | Defined within CD4+ T cells as: CD27-, CD45RA+, CD62L-/+ |
| Follicular CD4 T Cell | T follicular helper cells, defined within CD4+ T cells as: *CXCR5^hi^* (defined by gene expression); gene expression for *ICOS, CXCL13, PDCD1, IL21R, ASCL2, IL21, BCL6, and BTLA* were also employed to support the assignation of this population |
| T cell_CD4_Treg | Regulatory CD4+ T cells, defined within CD4+ T cells as: CD127^-/lo^, CD25^hi^; *FOXP3+* |
| **CD8+ T Cells** | **Defined within T Cells as CD4-, CD8+** |
| Naïve CD8 T Cell | Defined within CD8+ T cells as: CD27+, CD45RA+, CD62L+ |
| Central Memory  CD8 T Cell | Central memory CD8+ T cells, defined within CD8+ T cells as: CD27+, CD45RA^-/lo^, CD62L+, CD28+ |
| Transitional Memory  CD8 T Cell | Defined within CD8+ T cells as: CD27+, CD45RA^-/lo^, CD62L-, CD28+ |
| Effector Memory  CD8 T Cell | Defined within CD8+ T cells as: CD27-, CD45RA^-/lo^, CD62L-/+, CD28-/+ |
| Early Effector  CD8 T Cell | Defined within CD8+ T cells as: CD27+, CD45RA+, CD62L-/+, CD28^-/lo^; *GZMB^hi^* |
| Terminal Effector  CD8 T Cell | Defined within CD8+ T cells as: CD27-, CD45RA+, CD62L-/+ |
| Antigen Specific  CD8 T Cell | Defined within CD8+ T cells as: CD45RA- DR+, *MKI67+* |
|  |  |
| **Other Lymphocytes** |  |
| Innate Lymphoid Cell (ILC) | Defined within leukocytes as: *PTPRC+*, CD3-, CD11c-, CD14-, CD16-, CD19-, *FCER1A-, IL3RA-, CLEC9A-*, CD127+ |
| Natural Killer  (NK) Cell | Defined within leukocytes as: *PTPRC+,* CD3-, CD19-, CD14-, CD56+/++,  CD16-/+, CD45RA+ |
| NK Cell (CD56 bright) | Defined within leukocytes as: *PTPRC+*, CD3-, CD19-, CD14-, CD56++, CD16^-/lo^, CD45RA+ |
| NK Cell (CD56 dim) | Defined within leukocytes as: *PTPRC+*, CD3-, CD19-, CD14-, CD56+, CD16+, CD45RA+ |
|  |  |
| **Myeloid Cells** | **All leukocytes excluding Lymphoid Cells (B cells, T cells, NK cells, ILCs)** |
| Macrophages | Defined based on the expression of: HLA-DR+, CD11c+, and/or CD14+, CD19-, CD3-, CD56- and the expression of macrophage-related genes: *MCEMP1, PPARG, MRC1, CD68, LYZ, C1QA, and C1QB* in the absence of expression of monocytes and dendritic cell specific-genes |
| Mast Cells | Defined based on the expression of: *TPSAB1, TPSB2, CPA3, MS4A2, KIT, CLU, SLC18A2, GATA2, VWA5A* |
| Neutrophils | Defined based on the expression of: CD16lo/+, CD11c+, HLA-DR-, CD56-, CD3-, CD19-, CD62L-/+, *CXCR2+, PTGS2+, MME+* |
| Classical Monocytes | Defined based on the expression of: HLA-DR+, CD14+, CD16-, CD19- CD3- CD56- and the expression of monocyte-related genes: *CD86, IL1B, CD300E* in absence of expression of macrophage and dendritic cell specific-genes |
| Conventional Myeloid Dendritic Cell (cDC1) | CD141+ myeloid dendritic cells defined based on expression of: HLA-DR+ CD11c+, CD14^-/lo^, CD19-, CD3- and the expression of: *XCR1, CLEC9A, TBHD, CADM1, CST3* |
| Conventional Myeloid Dendritic Cell (cDC2) | CD1c+ myeloid dendritic cells defined based on expression of: HLA-DR+ CD11c+, CD14-, CD19-, CD3- and the expression of: *CLEC10A, CD1C, CD1A, FCER1A, CD207* |
| Plasmacytoid Dendritic Cell (pDC) | Defined based on the expression of HLA-DR+, CD11c-, CD14-, CD19-, CD3- and the expression of: *LILRA4, IRF7, IRF8, PACSIN1, TCF4, IL3RA, GZMB* |
|  |  |
| **NON-LEUKOCYTES** | **Defined as *PTPRCneg,* CD3-, CD19-, CD16-, CD14-** |
| Endothelial Cells | Defined based on the expression of endothelial cell markers: *TBX1, FLT1, PECAM1, VCAM1, CLDN5, VWF, FCN3, ACKR1* |
| Tumor Cells | Defined based on the expression of epithelial cell markers (mostly *EPCAM)*: *EPCAM, KRT17, KRT6A, AKR1C1* |
| Fibroblasts | Defined based on the expression of fibroblast markers: *LUM, MMP2, COL1A1, COL1A2, DCN* |
| Smooth Muscle Cells | Defined based on the expression of smooth muscle markers: *ACTA2, MYH11, CNN1, NOTCH3, DES* |
