# Supplementary material for: Pulsed Electric Field Ablation Reprograms Tumor Immunity and Stimulates Germinal Center Formation in Tertiary Lymphoid Structures in Patients with Non–Small Cell Lung Cancer
Source: Clin Cancer Res. 2026 Jun 17;32(17):3830–48. doi: 10.1158/1078-0432.CCR-25-3847 (PMC13530995; doi:10.1158/1078-0432.CCR-25-3847)
Supplement: Supplementary file 5 [file ccr-25-3847_supplementary_table_s4_suppts4.docx]

| **Supplementary Table 4. Flow Cytometry Panel Antibodies** | | | | |
| --- | --- | --- | --- | --- |
| **Marker** | **Antibody** | **Source** | **Catalog Number** | **Clone** |
| CCR4 | PE Cy7 | BioLegend | 359410 | L291H4 |
| CCR6 | BB700 | BD | 566477 | 11A9 |
| CCR10 | BUV395 | BD | 565322 | 1B5 |
| CD1c | BB515 | BD | 565054 | F10/21A3 |
| CD14 | APC H7 | BD | 641394 | MOP9 |
| CD16 | BUV496 | BD | 612944 | 3G8 |
| CD19 | PE Cy7 | Beckman Coulter | IM3628 | J3-119 |
| CD127 | BUV737 | BD | 612794 | HIL7RM21 |
| CD141 | BV421 | BD | 565321 | 1A4 |
| CD192 | BV605 | BioLegend | 357214 | K036C2 |
| CD20 | PE | BD | 555623 | 2H7 |
| CD25 | VioBright FITC | Miltenyi | 130-113-283 | 4E3 |
| CD27 | BV421 | BD | 562513 | M-T271 |
| CD28 | PerCP Cy5.5 | Biolegend | 302922 | CD28.2 |
| CD3 | BV605 | BD | 563852 | UCTH1 |
| CD33 | PE Cy7 | BD | 333952 | P67.6 |
| CD34 | PE | BD | 555822 | 581 |
| CD38 | APC H7 | BD | 656646 | HB7 |
| CD300e | APC | ImmunoStep | IREM2A-100T | UP-H2 |
| CD303 | APC | Miltenyi | 130-113-190 | AC144 |
| CD4 | APC H7 | BD | 641398 | SK3 |
| CD45 | AF700 | BD | 560566 | HI30 |
| CD45RA | BV510 | BD | 563031 | HI100 |
| CD5 | PerCP Cy5.5 | BD | 341109 | L17F12 |
| CD5 | BV510 | BD | 563381 | UCHT2 |
| CD56 | APC Vio770 | Miltenyi | 130-114-548 | REA196 |
| CD57 | FITC | BD | 333169 | HNK1 |
| CD62L | BV650 | BioLegend | 304860 | DREG56 |
| CD8 | BUV395 | BD | 563795 | RPA-T8 |
| CXCR3 | PE | BD | 557185 | 1C6/CXCR3 |
| CXCR5 | APC | Miltenyi | 130-122-926 | REA103 |
| FceR1 | PE | ImmunoStep | 1399990356 | AER-37 (CRA1) |
| HLA DR | BUV395 | BD | 564040 | G46-6 |
| IgD | APC | BD | 561303 | IA6-2 |
| IgM | BV510 | BioLegend | 314522 | MHM-88 |
| PD1 | BV785 | BioLegend | 329930 | EH12.2H7 |
| PD-L1 | PE eFluor610 | ThermoFisher | 61-5983-42 | MIH1 |
| Slan | PE | Miltenyi | 130-119-867 | DD-1 |
| TCRgd | PE Cy7 | BD | 655410 | 11F2 |
| Viability | ZombieNIR | BioLegend | 423106 |  |
